# Semiparametric marginal regression for clustered competing risks data with missing cause of failure

**DOI:** 10.1093/biostatistics/kxac012

**Published:** 2022-04-12

**Authors:** Wenxian Zhou, Giorgos Bakoyannis, Ying Zhang, Constantin T Yiannoutsos

**Affiliations:** Department of Biostatistics and Health Data Science, Indiana University, 410 West 10th Street, Suite 3000, Indianapolis, IN 46202, USA; Department of Biostatistics and Health Data Science, Indiana University, 410 West 10th Street, Suite 3000, Indianapolis, IN 46202, USA; Department of Biostatistics, University of Nebraska Medical Center 42nd and Emile, Omaha, NE 68198, USA; Department of Biostatistics and Health Data Science, Indiana University, 410 West 10th Street, Suite 3000, Indianapolis, IN 46202, USA

**Keywords:** Clustered data, Competing risks, Informative cluster size, Missing cause of failure

## Abstract

Clustered competing risks data are commonly encountered in multicenter studies. The analysis of such data is often complicated due to informative cluster size (ICS), a situation where the outcomes under study are associated with the size of the cluster. In addition, the cause of failure is frequently incompletely observed in real-world settings. To the best of our knowledge, there is no methodology for population-averaged analysis with clustered competing risks data with an ICS and missing causes of failure. To address this problem, we consider the semiparametric marginal proportional cause-specific hazards model and propose a maximum partial pseudolikelihood estimator under a missing at random assumption. To make the latter assumption more plausible in practice, we allow for auxiliary variables that may be related to the probability of missingness. The proposed method does not impose assumptions regarding the within-cluster dependence and allows for ICS. The asymptotic properties of the proposed estimators for both regression coefficients and infinite-dimensional parameters, such as the marginal cumulative incidence functions, are rigorously established. Simulation studies show that the proposed method performs well and that methods that ignore the within-cluster dependence and the ICS lead to invalid inferences. The proposed method is applied to competing risks data from a large multicenter HIV study in sub-Saharan Africa where a significant portion of causes of failure is missing.

## 1. Introduction

Clustered competing risks data are commonly encountered in multicenter studies (Zhou *and others,* 2012; Diao and Zeng, 2013). An important feature of such data is that the outcomes of individuals from the same cluster are typically dependent and, thus, the standard assumption of independence is violated (Balan and Putter, 2019; Bakoyannis, 2021). Therefore, standard methods for competing risks data are not applicable in the presence of clustering. In addition, cluster size is often informative, in the sense that the outcomes under study are associated with cluster size (Pavlou *and others,* 2013; Seaman *and others,* 2014; Cong *and others,* 2007). An example of a setting with informative cluster size (ICS) is a study about dental health outcomes. In such studies, dental health outcomes are related to the total number of teeth (cluster size) in a given person (Williamson *and others,* 2008). With ICS, the standard methods for clustered data lead to bias, since larger clusters contribute a larger portion of observations in the sample and, thus, have a larger influence on the parameter estimates. Furthermore, the cause of failure is frequently incompletely observed in real-world settings due to nonresponse/missingness or by the study design (Bakoyannis *and others,* 2020). A complete case analysis that discards observations with missing event types is expected to lead to biased inference (Lu and Tsiatis, 2001; Bakoyannis *and others,* 2010).

This work is motivated by a large multicenter HIV cohort study conducted by the East Africa Regional Consortium of the International Epidemiology Databases to Evaluate AIDS (EA-IeDEA). A major goal of the study was to evaluate HIV healthcare clinics in East Africa and study two important outcomes in HIV care: (i) disengagement from care and (ii) death while in care (i.e., prior to disengagement). In this study, patients who received antiretroviral treatment (ART) at the same clinic are expected to have correlated outcomes. In addition, the number of patients in the clinic is expected to be associated with the outcomes of interest (ICS) since clinics with more patients are typically better staffed and are expected to provide better care. Furthermore, there is a severe death under-reporting issue in sub-Saharan Africa. Given that unreported deaths are incorrectly classified as disengagements from care, as patients who do not show up in care for a scheduled clinic visit are automatically classified as disengagers, the latter issue leads to outcome misclassification. To deal with this, EA-IeDEA implemented a double-sampling design where a small subset of patients who were lost to the clinic were intensively outreached in the community and their vital status was actively ascertained. This double-sampling design transformed the misclassification problem into a missing data problem by the study design, where the cause of failure (i.e., death while in care or disengagement) was unknown for the nonoutreached lost patients.

There are two main classes of models for dealing with the within-cluster dependence issue for survival data. One is frailty models (Clayton and Cuzick, 1985; Hougaard, 1986; Liu *and others,* 2011), which specify explicitly the within-cluster dependence via random effects and provide cluster-specific inference. Such models typically impose assumptions about the structure of the within-cluster dependence and the distribution of the random effects and tend to be computationally intensive. Under this class of models, Katsahian *and others* (2006) proposed a frailty proportional hazards model for the subdistribution of a competing risk, while Scheike *and others* (2010) proposed a semiparametric random-effects model for competing risks data where the interest is on a particular cause of failure. The other class of models is marginal models (Wei *and others,* 1989; Liang *and others,* 1993; Cai and Prentice, 1997; Spiekerman and Lin, 1998; Cai *and others,* 2000). These models do not rely on assumptions regarding the dependence structure and have a population-averaged interpretation. Following this idea, Zhou *and others* (2012) proposed a marginal version of the Fine–Gray model (Fine and Gray, 1999) for population-averaged analysis of clustered competing risks.

In settings with ICS, there are typically two populations of interest: (i) the population of *all cluster members* (ACM) and (ii) the population of *typical cluster members* (TCM) (Seaman *and others,* 2014). The former population includes all the observations from all the clusters, while the latter one concerns a single (randomly selected) observation from each cluster. As a result, the ACM population is dominated by the larger clusters, while all clusters are equally represented in the TCM population. In the context of survival analysis, the ICS issue has been addressed via a within-cluster resampling method (Cong *and others,* 2007) and a weighted score function approach (Williamson *and others,* 2008), where the weights are equal to the inverse of the cluster size. Both methods provide inference for the TCM population.

The issue of missing cause of failure with independent competing risks data has received considerable attention in the literature (Goetghebeur and Ryan, 1995; Lu and Tsiatis, 2001; Craiu and Duchesne, 2004; Gao and Tsiatis, 2005; Lu and Liang, 2008; Bakoyannis *and others,* 2010; Hyun *and others,* 2012; Bordes *and others,* 2014; Nevo *and others,* 2018; Bakoyannis *and others,* 2020). Recently, Bakoyannis *and others* (2020) proposed a unified framework for semiparametric regression and risk prediction for competing risks data with missing at random (MAR) cause of failure, under the proportional cause-specific hazards model. Unlike previous methods, the approach by Bakoyannis *and others* (2020) provides inference for both regression and functional parameters such as the cumulative incidence function. The latter quantity is the key for risk prediction in modern medicine. Moreover, simulation studies have shown that the approach by Bakoyannis *and others* (2020) provides substantially more efficient regression parameter estimates compared to augmented inverse probability weighting estimators (Gao and Tsiatis, 2005; Hyun *and others,* 2012) and the multiple-imputation estimator (Lu and Tsiatis, 2001). However, all the aforementioned methods did not consider a potential within-cluster dependence and are thus expected to lead to invalid inferences with clustered data. To the best of our knowledge, only Lee *and others* (2017) have addressed the issue of analyzing clustered competing risks data with the missing cause of failure. Lee *and others* (2017) proposed a frailty proportional cause-specific hazards model along with a hierarchical likelihood approach for estimation. Nevertheless, this approach does not allow for ICS, it imposes strong assumptions regarding the within-cluster dependence and the distribution of the frailty, which may be violated in practice, and does not provide inference for the infinite-dimensional parameters such as the cumulative incidence function. In addition, the method provides cluster-specific inference and not population-averaged inference which is more scientifically relevant in many applications, including our motivating HIV study.

To the best of our knowledge, there is no general method for marginal competing risks analysis based on data with (i) missing causes of failure, (ii) within-cluster dependence, and (iii) ICS. Nevertheless, all these complications are encountered in the competing risks data from our motivating EA-IeDEA study, as described above. The novelty of this work is that it addresses all these practically important complications via a rigorous approach. To this end, we consider the semiparametric marginal proportional cause-specific hazards model and propose a maximum partial pseudolikelihood estimator under a MAR assumption. Even though our methodology provides inference for the TCM population, it can be very easily modified to provide inference for the ACM population, as described in Section 5. The proposed method does not impose assumptions regarding the within-cluster dependence and allows for ICS. Moreover, the method can be easily implemented using the R code presented in the Supplementary material available at *Biostatistics* online or our R functions available at https://github.com/wz11/ClusteredMPPLE. The proposed estimators are shown to be strongly consistent and asymptotically normal. Closed-form variance estimators are provided and a rigorous methodology for the calculation of simultaneous confidence bands for the infinite-dimensional parameters is proposed. Simulation studies show that the method performs well and that the previously proposed method for missing causes of failure by Bakoyannis *and others* (2020), which ignores the within-cluster dependence and the potential ICS, leads to invalid inferences. Finally, the method is applied to the data from the EA-IeDEA study.

## 2. Methodology

### 2.1. Notation and assumptions

Let $i=1,2, \ldots, n$ index the $n$ clusters in the study and $j=1,2, \ldots, M_{i}$ index the subjects in the $i$th cluster. Also, let $T_{i j}$ and $U_{i j}$ denote the failure and right censoring times for the $j$th subject in the $i$th cluster. The corresponding observed counterparts are the minimum of the event or censoring times $X_{i j}=T_{i j} \wedge U_{i j}$ and the failure (from any cause) indicator $\Delta_{i j}=I(T_{i j} \leq U_{i j})$. Here, we consider the finite observation interval $[0, \tau]$, for an arbitrary $\tau<\infty$. Suppose that there are $k$ competing causes of failure, with $k<\infty$, and let $C_{i j} \in\{1,2, \ldots, k\}$ denote the cause of failure for the $j$th subject in the $i$th cluster. For the sake of generality, cluster size $M$ is assumed to be random and informative, in the sense that there is an association between the event time and/or cause of failure and $M$. However, our proposed methodology applies trivially to simpler situations with noninformative or fixed cluster size. To incorporate missingness in the cause of failure, we define the missing indicator $R_{i j}$, with $R_{i j}=1$ indicating that the cause of failure for the $j$th subject in the $i$th cluster is observed, and $R_{i j}=0$ otherwise. As in previous works on missing cause of failure (Bakoyannis *and others,* 2020), we consider the situation where right censoring status is always observed, that is if $\Delta_{i j}=0$ then $R_{i j}=1$. The cause of failure $C_{i j}$ is only observed when both $\Delta_{i j}=1$ and $R_{i j}=1$. Let $\epsilon_{i j}=\Delta_{i j} R_{i j} C_{i j}$ be the observed cause of failure, with $\epsilon_{i j}=0$ denoting the cause of failure is missing or censored. The vector of covariates of scientific interest is denoted by $\boldsymbol{Z}_{i j}\in\mathbb{R}^p$. In addition, let $\boldsymbol{A}_{i j} \in \mathbb{R}^{q}$ denote a vector of auxiliary variables, which may not be of scientific interest, but may be related to the probability of missingness. It has been argued that such auxiliary covariates can be used to make the MAR assumption more plausible in practice (Lu and Tsiatis, 2001; Nevo *and others,* 2018; Bakoyannis *and others,* 2019, 2020). As usual, $(T_{ij}, C_{ij})$ and $U_{ij}$ are assumed independent given $\boldsymbol{Z}_{ij}$. In addition, $(T_{ij}, C_{ij}, U_{ij})$ are assumed independent across clusters conditionally on $\boldsymbol{Z}_{ij}$. However, within cluster $i$, $(T_{i j}, C_{i j})$, $j=1, \ldots, M_{i}$, are allowed to be dependent given $\boldsymbol{Z}_{ij}$, with an arbitrary dependence structure. Similarly, the right censoring times $U_{i j}$ may be dependent within cluster $i$. The observed data are $n$ i.i.d. copies of $\boldsymbol{D}_{i}=(\boldsymbol{X}_{i}, \boldsymbol{\Delta}_{i}, \boldsymbol{\epsilon}_{i}, \boldsymbol{Z}_{i}, \boldsymbol{A}_{i}, \boldsymbol{R}_{i}, M_{i})$, $i=1, \ldots, n$, where $\boldsymbol{X}_{i}=\{X_{i j}\}_{j=1}^{M_{i}}$, $\boldsymbol{\Delta}_{i}=\{\Delta_{i j}\}_{j=1}^{M_{i}}$, $\boldsymbol{\epsilon}_{i}=\{\epsilon_{i j}\}_{j=1}^{M_{i}}$, $\boldsymbol{Z}_{i}=\{\boldsymbol{Z}_{i j}\}_{j=1}^{M_{i}}$, $\boldsymbol{A}_{i}=\{\boldsymbol{A}_{i j}\}_{j=1}^{M_{i}}$, and $\boldsymbol{R}_{i}=\{R_{i j}\}_{j=1}^{M_{i}}$. To facilitate the presentation of the proposed estimator and its properties, we define the counting process $N_{i j}(t)=I(X_{i j} \leq t, \Delta_{i j}=1)$ and at-risk process $Y_{i j}(t)=I(X_{i j} \geq t)$. Additionally, we define the cause-specific counting process as $N_{i j l}(t)=I(X_{i j} \leq t, \Delta_{i j l}=1)=\Delta_{i j l} N_{i j}(t)$, where $\Delta_{i j l}=I(C_{i j}=l, \Delta_{i j}=1)$, for $l=1, \ldots k$.

Letting $\boldsymbol{W}_{i j}=(X_{ij}, \boldsymbol{Z}_{i j}, \boldsymbol{A}_{i j})$, we impose the MAR assumption $P(R_{i j}=1|C_{i j}, \Delta_{i j}=1, \boldsymbol{W}_{i j})=P(R_{i j}=1|\Delta_{i j}=1, \boldsymbol{W}_{i j})$. This assumption is equivalent to

\begin{equation*} P(C_{i j}=l|R_{i j},\Delta_{i j}=1, \boldsymbol{W}_{i j})=P(C_{i j}=l|\Delta_{i j}=1,\boldsymbol{W}_{i j})\equiv\pi_l(\boldsymbol{W}_{i j},\boldsymbol{\gamma}_{0}), \quad l=1, \ldots, k,\end{equation*}

where $\pi_l(\boldsymbol{W}_{i j},\boldsymbol{\gamma}_{0})$ is the marginal probability of the failure cause $l$ given $\boldsymbol{W}_{i j}$, for a non-right-censored observation, and $\boldsymbol{\gamma}_{0}$ is assumed to be a finite-dimensional parameter. In Section 2.3, we provide a goodness-of-fit approach for evaluating the appropriateness of this model in practice.

### 2.2. Estimation approach

In this work, we provide estimators and inference methodology for both marginal cause-specific hazards and cumulative incidence functions. The covariate-specific marginal cause-specific hazards are defined as

\begin{equation*} \lambda_{l}\left(t ; \boldsymbol{z}\right)=\lim_{h \rightarrow 0} \frac{P\left(t \leq T_{i j}<t+h, C_{i j}=l \mid T_{i j} \geq t, \boldsymbol{Z}_{i j}=\boldsymbol{z}\right)}{h}, \quad l=1, \ldots k,\end{equation*}

and the covariate-specific marginal cumulative incidence functions are defined as

(2.1) \begin{equation*} F_{l}\left(t ; \boldsymbol{z}\right)=P\left(T_{i j} \leq t, C_{i j}=l \mid \boldsymbol{Z}_{i j}=\boldsymbol{z}\right)=\int_{0}^{t} \exp \left[-\sum_{l=1}^{k} \Lambda_{l}\left(s ; \boldsymbol{z}\right)\right] \lambda_{l}\left(s ; \boldsymbol{z}\right) ds, \label{cif} \end{equation*}

for $l=1,\ldots k$ and $t\in[0,\tau]$, where $\Lambda_{l}(t ; \boldsymbol{z})=\int_{0}^{t} \lambda_{l}(s ; \boldsymbol{z}) {\rm d}s$ is the covariate-specific cumulative hazard for the $l$th cause of failure. Here, we adopt the marginal proportional cause-specific hazards model $\lambda_{l}\left(t ; \boldsymbol{z}\right)=\lambda_{0,l}(t) \exp \left(\boldsymbol{\beta}_{0,l}^{T} \boldsymbol{z}\right)$, $l=1, \ldots, k$, where $\lambda_{0,l}(t)$ is the $l$th unspecified baseline cause-specific hazard function.

When there are no missing causes of failure (i.e., $R_{ij}=1$ for $j=1,\ldots,M_i$ and $i=1,\ldots,n$), estimation for clustered competing risks data can be performed, under the working independence assumption, using the logarithm of the weighted partial likelihood for $\boldsymbol{\beta}=(\boldsymbol{\beta}_{1}, \ldots, \boldsymbol{\beta}_{k})$

(2.2) \begin{eqnarray*} pl_{n}(\boldsymbol{\beta})=\sum_{l=1}^{k} \sum_{i=1}^{n} \frac{1}{M_{i}} \sum_{j=1}^{M_{i}} \int_{0}^{\tau}\left[\boldsymbol{\beta}_{l}^{T} \boldsymbol{Z}_{i j}-\log \left\{\sum_{p=1}^{n} \frac{1}{M_{p}} \sum_{q=1}^{M_{p}} Y_{p q}(t) \exp \left(\boldsymbol{\beta}_{l}^{T} \boldsymbol{Z}_{p q}\right)\right\}\right] {\rm d} N_{i j l}(t).\quad \label{loglik} \end{eqnarray*}

This can be seen as the competing risks analog of the weighted log-partial likelihood by Cong *and others* (2007), where the contribution of each subject is weighted by the inverse of the corresponding cluster size to account for ICS.

When cause of failure is missing for some individuals, the weighted log-partial likelihood (2.2) cannot be evaluated for the observations with a missing cause of failure. For such situations, we propose a weighted partial pseudolikelihood estimator for $\boldsymbol{\beta}$ which replaces the unobserved cause-specific counting processes with their conditional expectation given the observed data $\boldsymbol{D}_{ij}$. These conditional expectations are equal to $\tilde{N}_{i j l}\left(t ; \boldsymbol{\gamma}_{0}\right)\equiv E\{N_{i j l}(t)|\boldsymbol{D}_{ij}\}=\left\{R_{i j} \Delta_{i j l}+\left(1-R_{i j}\right) \pi_{l}\left(\boldsymbol{W}_{i j}, \boldsymbol{\gamma}_{0}\right)\right\} N_{i j}(t)$. The resulting logarithm of the expected partial pseudolikelihood conditional on the observed data $\{\boldsymbol{D}_{i j}\}_{i=1, \ldots n ; j=1, \ldots M_{i}}$ is

\begin{equation*} Q_n(\boldsymbol{\beta};\boldsymbol{\gamma}_0)= \sum_{l=1}^{k} \sum_{i=1}^{n} \frac{1}{M_{i}} \sum_{j=1}^{M_{i}} \int_{0}^{\tau}\left[\boldsymbol{\beta}_{l}^{T} \boldsymbol{Z}_{i j}-\log \left\{\sum_{p=1}^{n} \frac{1}{M_{p}} \sum_{q=1}^{M_{p}} Y_{p q}(t) \exp \left(\boldsymbol{\beta}_{l}^{T} \boldsymbol{Z}_{p q}\right)\right\}\right] {\rm d} \tilde{N}_{i j l}\left(t ; \boldsymbol{\gamma}_{0}\right)\!.\end{equation*}

Our estimation approach consists of two steps. The first step involves the estimation of the unknown parameter $\boldsymbol{\gamma}_{0}$ in $Q_n(\boldsymbol{\beta};\boldsymbol{\gamma}_0)$. This can be achieved by fitting the marginal binary or multinomial logistic model on the complete cases using generalized estimating equations weighted by the inverse of the cluster size (Seaman *and others,* 2014) and under a working independence assumption. The second step of our approach involves the estimation of $\boldsymbol{\beta}_0$ using the partial pseudoscore function

\begin{equation*} \boldsymbol{G}_{n,l}\left(\boldsymbol{\beta}; \hat{\boldsymbol{\gamma}}_n\right)=\frac{1}{n} \sum_{i=1}^{n} \frac{1}{M_{i}} \sum_{j=1}^{M_{i}} \int_{0}^{\tau}\left\{\boldsymbol{Z}_{i j}-\boldsymbol{E}_{n}\left(t, \boldsymbol{\beta}_{l}\right)\right\} d \tilde{N}_{i j l}\left(t ; \hat{\boldsymbol{\gamma}}_n\right), \quad l=1, \ldots, k,\end{equation*}

with $\hat{\boldsymbol{\gamma}}_n$ being the estimate of the unknown parameter $\boldsymbol{\gamma}_0$ (computed in the first step), where

\begin{equation*} \boldsymbol{E}_{n}\left(t, \boldsymbol{\beta}_{l}\right)=\frac{\sum_{p=1}^{n} \frac{1}{M_{p}} \sum_{q=1}^{M_{p}} Y_{p q}(t) \exp (\boldsymbol{\beta}_{l}^{T} \boldsymbol{Z}_{p q}) \boldsymbol{Z}_{p q}}{\sum_{p=1}^{n} \frac{1}{M_{p}} \sum_{q=1}^{M_{p}} Y_{p q}(t) \exp (\boldsymbol{\beta}_{l}^{T} \boldsymbol{Z}_{p q})}.\end{equation*}

The estimators $\hat{\boldsymbol{\beta}}_{n,l}$ are the solutions to $\boldsymbol{G}_{n,l}(\hat{\boldsymbol{\beta}}_{n,l},\hat{\boldsymbol{\gamma}}_n)=\boldsymbol{0}$, $l=1, \ldots, k$. The Breslow-type estimator for the marginal cause-specific baseline cumulative hazard function is

\begin{equation*} \hat{\Lambda}_{n,l}(t)=\sum_{i=1}^{n} \frac{1}{M_{i}} \sum_{j=1}^{M_{i}} \int_{0}^{t} \frac{{\rm d} \tilde{N}_{i j l}\left(u ; \hat{\boldsymbol{\gamma}}_n\right)}{\sum_{p=1}^{n} \frac{1}{M_{p}} \sum_{q=1}^{M_{p}} Y_{p q}(u) \exp \left(\hat{\boldsymbol{\beta}}_{n,l}^{T} \boldsymbol{Z}_{p q}\right)}, \ \ \ \ t\in[0,\tau], \ \ l=1, \ldots, k.\end{equation*}

Based on this estimator, the marginal covariate-specific cumulative incidence function can be estimated by

\begin{equation*} \hat{F}_{n,l}(t ; \boldsymbol{z}_{0})=\int_{0}^{t} \exp \left[-\sum_{l=1}^{k} \hat{\Lambda}_{n,l}(u-; \boldsymbol{z}_{0})\right] {\rm d} \hat{\Lambda}_{n,l}(u ; \boldsymbol{z}_{0}),\end{equation*}

where $\hat{\Lambda}_{n,l}(t;\boldsymbol{z}_{0})=\hat{\Lambda}_{n,l}(t) \exp (\hat{\boldsymbol{\beta}}_{n,l}^{T} \boldsymbol{z}_{0})$. We must note that the uncertainty in the estimate $\hat{\boldsymbol{\gamma}}_n$ is properly incorporated into all closed-form variance estimators provided in Section 2.3.

### 2.3. Asymptotic properties

Here, we state the main theorems for the asymptotic properties of the proposed estimators $\hat{\boldsymbol{\beta}}_{n,l}$, $\hat{\Lambda}_{n,l}(t)$ and $\hat{F}_{n,l}(t ; \boldsymbol{z}_{0})$. The proofs of these theorems are provided in the Supplementary material available at *Biostatistics* online. A key assumption in this work is that the model for $\pi_{l}(\boldsymbol{W}_{i j}, \boldsymbol{\gamma}_{0})$ is correctly specified. This assumption can be evaluated using the residual processes $E\left[\frac{1}{M}\sum_{j=1}^{M} R_{j}\{N_{j l}(t)-\pi_l(\boldsymbol{W}_{ j},\boldsymbol{\gamma}_{0})N_{j}(t)\}\right]$, $l=1, \ldots, k-1$, $t \in [0,\tau]$, which can be estimated by $\frac{1}{n} \sum_{i=1}^{n} \frac{1}{M_{i}}\sum_{j=1}^{M_{i}} R_{i j}\{N_{i j l}(t)-\pi_l(\boldsymbol{W}_{i j},\hat{\boldsymbol{\gamma}}_{n})N_{ij}(t)\}$. If the model is correctly specified, the cumulative residual processes are all equal to 0 for $t \in [0,\tau]$. A goodness-of-fit test can be conducted using the simulation-based approach by Pan and Lin (2005). A graphical evaluation of the goodness of fit can also be performed by plotting the observed residual process and the 9$\%$ simultaneous confidence band around the line $f(t)=0$, $t \in [0,\tau]$ (Bakoyannis *and others,* 2019, 2020). Theorem 2.1 states the consistency of the proposed estimators.

Theorem 2.1 Under the assumptions in Section 2.1 and regularity conditions C1–C7 in the Supplementary material available at *Biostatistics* online

\begin{equation*} \sum_{l=1}^{k}\left\{\|\hat{\boldsymbol{\beta}}_{n,l}-\boldsymbol{\beta}_{0,l}\|+\sup_{ t\in [0,\tau]}| \hat{\Lambda}_{n,l}(t)-\Lambda_{0,l}(t) |\right\} \rightarrow_{as^{*}} 0, \ \ {\rm as} \ \ n\rightarrow\infty.\end{equation*}

A corollary of Theorem 2.1 is the strong uniform consistency of $\hat{F}_{n,l}(t ; \boldsymbol{z}_{0})$, $l=1,\ldots,k$, that is $\sum_{l=1}^{k}\sup_{ t\in [0,\tau]}|\hat{F}_{n,l}(t ; \boldsymbol{z}_{0})-F_{0,l}(t ; \boldsymbol{z}_{0})| \rightarrow_{a s^{*}} 0$. Theorem 2.2 provides the asymptotic distribution for the estimated regression coefficient $\hat{\boldsymbol{\beta}}_{n, l}$. This theorem is useful for conducting hypothesis testing and calculating confidence intervals (CIs) for $\boldsymbol{\beta}_{0,l}$, $l=1, \ldots, k$.

Theorem 2.2 Under the assumptions in Section 2.1 and regularity conditions C1–C7 in the Supplementary material available at *Biostatistics* online, we have that

\begin{equation*} \sqrt{n}(\hat{\boldsymbol{\beta}}_{n, l}-\boldsymbol{\beta}_{0, l})=n^{-1/2} \sum_{i=1}^{n}\left\{M_{i}^{-1} \sum_{j=1}^{M_{i}}\left(\boldsymbol{\psi}_{i j l}+\boldsymbol{R}_{l}\boldsymbol{\omega}_{i j}\right)\right\}+o_{p}(1), \ \ \ \ l=1, \ldots, k,\end{equation*}

where explicit formulas for $\boldsymbol{\psi}_{i j l}$, $\boldsymbol{R}_{l}$, and $\boldsymbol{\omega}_{i j}$ are provided in the Supplementary material available at *Biostatistics* online.

By Theorem 2.2, $\sqrt{n}(\hat{\boldsymbol{\beta}}_{n, l}-\boldsymbol{\beta}_{0, l}) \rightarrow_{d} N(\boldsymbol{0}, \boldsymbol{\Sigma}_{l})$, where $ \boldsymbol{\Sigma}_{l}=E\{M^{-1} \sum_{j=1}^{M}(\boldsymbol{\psi}_{j l}+\boldsymbol{R}_{l}\boldsymbol{\omega}_{j})\}^{\otimes 2}$. The covariance matrix $\boldsymbol{\Sigma}_{l}$ can be consistently estimated using the empirical versions of the influence functions by $\hat{\boldsymbol{\Sigma}}_{l}=\frac{1}{n} \sum_{i=1}^{n} \left\{\frac{1}{M_{i}} \sum_{j=1}^{M_{i}}\left(\hat{\boldsymbol{\psi}}_{i j l}+\hat{\boldsymbol{R}}_{l}\hat{\boldsymbol{\omega}}_{i j}\right)\right\}^{\otimes 2}$. The empirical versions of the influence functions can be obtained by replacing expectations with sample averages over clusters and unknown parameters with their consistent estimates. Explicit formulas for the empirical versions of the influence functions are provided in the Supplementary material available at *Biostatistics* online.

Theorems 2.3 and 2.4 provide the weak convergence of $\hat{\Lambda}_{n,l}(t)$ and $\hat{F}_{n,l}(t ; \boldsymbol{z}_{0})$, respectively. Before providing them, we let $D[0,\tau]$ denote the space of right-continuous functions with left-hand limits defined on $[0, \tau]$, and $\{\xi_{i}\}_{i=1}^{n}$ be standard normal variables independent of the data.

Theorem 2.3 Under the assumptions in Section 2.1 and regularity conditions C1–C7 in the Supplementary material available at *Biostatistics* online, we have that

\begin{equation*} \sqrt{n}\left\{\hat{\Lambda}_{n, l}(t)-\Lambda_{0, l}(t)\right\}=\frac{1}{\sqrt{n}} \sum_{i=1}^{n}\left[ \frac{1}{M_{i}} \sum_{j=1}^{M_{i}} \left\{\phi_{i j l}(t)+\boldsymbol{R}_{l}^{*}(t) \boldsymbol{\omega}_{i j}\right\}\right]+o_{p}(1), \ \ \ \ l=1, \ldots, k, \ \ t\in[0,\tau],\end{equation*}

with the influence functions belonging to a Donsker class, and conditional on the observed data, $\hat{W}_{n, l}(\cdot)=n^{-1/2} \sum_{i=1}^{n}[ M_{i}^{-1} \sum_{j=1}^{M_{i}}\{\hat{\phi}_{i j l}(\cdot)+\hat{\boldsymbol{R}}_{l}^{*}(\cdot) \hat{\boldsymbol{\omega}}_{i j}\}]\xi_{i}$ converges weakly to the same limiting process as $W_{n, l}(\cdot)=\sqrt{n}\{\hat{\Lambda}_{n, l}(\cdot)-\Lambda_{0, l}(\cdot)\}$. Explicit formulas for $\phi_{i j l}(t)$, $\boldsymbol{R}_{l}^{*}(t)$, $\hat{\phi}_{i j l}(t)$, and $\hat{\boldsymbol{R}}_{l}^{*}(t)$ are provided in the Supplementary material available at *Biostatistics* online.

By Theorem 2.3, $\sqrt{n}\{\hat{\Lambda}_{n, l}(\cdot)-\Lambda_{0, l}(\cdot)\}\leadsto \mathbb{G}_{\Lambda_l}$ in $D[0,\tau]$, where $\mathbb{G}_{\Lambda_l}$ is a tight mean zero Gaussian process with covariance function $E[ M^{-1} \sum_{j=1}^{M} \{\phi_{j l}(t)+\boldsymbol{R}_{l}^{*}(t) \boldsymbol{\omega}_{j}\}][ M^{-1} \sum_{j=1}^{M} \{\phi_{j l}(s)+\boldsymbol{R}_{l}^{*}(s) \boldsymbol{\omega}_{j}\}]$, $t,s \in [0, \tau]$. A consistent estimator of the covariance function is

$$\frac{1}{n} \sum_{i=1}^{n}\left[\frac{1}{M_{i}} \sum_{j=1}^{M_{i}}\left\{\hat{\phi}_{i j l}(t)+\hat{\boldsymbol{R}}_{l}^{*}(t) \hat{\boldsymbol{\omega}}_{i j}\right\}\right]\left[\frac{1}{M_{i}} \sum_{j=1}^{M_{i}}\left\{\hat{\phi}_{i j l}(s)+\hat{\boldsymbol{R}}_{l}^{*}(s) \hat{\boldsymbol{\omega}}_{i j}\right\}\right], \quad t,s \in [0, \tau].$$

Calculation of CIs and bands can be performed using an appropriate continuously differentiable transformation to avoid negative limits (Lin *and others,* 1994). A standard choice is the transformation $g(x)=\log (x)$. According to the functional delta method, $\sqrt{n} q_{l}^{\Lambda}(t)[g\{\hat{\Lambda}_{n, l}(t)\}-g\{\Lambda_{0, l}(t)\}]$ is asymptotically equivalent to $B_{n, l}(t)=q_{l}^{\Lambda}(t) \dot{g}\{\hat{\Lambda}_{n, l}(t)\} W_{n, l}(t)$. Also, by Theorem 2.3, $B_{n, l}(t)$ is asymptotically equivalent to $\hat{B}_{n, l}(t)=q_{l}^{\Lambda}(t) \dot{g}\{\hat{\Lambda}_{n, l}(t)\} \hat{W}_{n, l}(t)$, where $q_{l}^{\Lambda}(t)$ is a weight function, with $t \in[t_{1}, t_{2}]$, $0 \leq t_{1} \leq t_{2} < \tau$. The choice $q_{l}^{\Lambda}(t)=\hat{\Lambda}_{n, l}(t)\{\hat{\sigma}_{\Lambda_{l}}(t)\}^{-1}$, where $\hat{\sigma}_{\Lambda_{l}}(t)$ is the square root of the estimated variance of $\hat{\Lambda}_{n, l}(t)$, gives the equal precision band (Nair, 1984); the choice $q_{l}^{\Lambda}(t)=\hat{\Lambda}_{n, l}(t)\{1+\hat{\sigma}^{2}_{\Lambda_{l}}(t)\}^{-1}$, provides a Hall–Wellner-type band (Hall and Wellner, 1980). Now, a $1-\alpha$ confidence band for $\Lambda_{0, l}(t)$ can be computed as $g^{-1}\left[g\left\{\hat{\Lambda}_{n, l}(t)\right\} \pm \frac{c_{\alpha}}{\sqrt{n} q_{l}^{\Lambda}(t)}\right]$, $t \in[t_{1}, t_{2}]$, where $c_{\alpha}$ is the $1-\alpha$ quantile of the distribution of $\sup _{t \in[t_{1}, t_{2}]}|\hat{B}_{n,l}(t)|$. This can be estimated using a large number of simulation realizations from the process $\hat{B}_{n,l}(\cdot)$, generated by repeatedly simulating sets of standard normal variables $\{\xi_i\}_{i=1}^n$ (Spiekerman and Lin, 1998). Since confidence bands tend to be unstable at earlier and later time points, where there are fewer observed events, we suggest the restriction of the confidence band domain $[t_{1}, t_{2}]$ to the 10th and 90th percentile of the event times.

Theorem 2.4 Under the assumptions in Section 2.1 and regularity conditions C1–C7 in the Supplementary material available at *Biostatistics* online, we have that

\begin{equation*} \sqrt{n}\left\{\hat{F}_{n,l}(t ; \boldsymbol{z}_{0})-F_{0,l}(t ; \boldsymbol{z}_{0})\right\}=\frac{1}{\sqrt{n}}\sum_{i=1}^{n} \left\{\frac{1}{M_{i}} \sum_{j=1}^{M_{i}}\phi_{i j l}^{F}(t ; \boldsymbol{z}_{0})\right\}+o_{p}(1), \ \ \ \ l=1, \ldots, k, \ \ t\in[0,\tau],\end{equation*}

with the influence functions belonging to a Donsker class, and conditionally on the observed data, $\hat{W}_{n, l}^{F}(\cdot ; \boldsymbol{z}_{0})=n^{-1/2} \sum_{i=1}^{n} \{ M_{i}^{-1} \sum_{j=1}^{M_{i}} \hat{\phi}_{i j l}^{F}(\cdot ; \boldsymbol{z}_{0})\} \xi_{i}$ converges weakly to the same limiting process as $W_{n, l}^{F}(\cdot ; \boldsymbol{z}_{0})=\sqrt{n}\{\hat{F}_{n,l}(\cdot ; \boldsymbol{z}_{0})-F_{0,l}(\cdot ; \boldsymbol{z}_{0})\}$. Explicit formulas for $\phi_{i j l}^{F}(t; \boldsymbol{z}_{0})$ and $\hat{\phi}_{i j l}^{F}(t; \boldsymbol{z}_{0})$ are given in the Supplementary material available at *Biostatistics* online.

By Theorem 2.4, $\sqrt{n}\{\hat{F}_{n,l}(\cdot ; \boldsymbol{z}_{0})-F_{0,l}(\cdot ; \boldsymbol{z}_{0})\}\leadsto \mathbb{G}_{F_l}$ in $D[0,\tau]$, where $\mathbb{G}_{F_l}$ is a tight mean zero Gaussian process with covariance function $E\{ M^{-1} \sum_{j=1}^{M} \phi_{j l}^{F}(t ; \boldsymbol{z}_{0})\}\{ M^{-1} \sum_{j=1}^{M} \phi_{j l}^{F}(s ; \boldsymbol{z}_{0})\}$. A consistent estimator for the covariance function is $\frac{1}{n} \sum_{i=1}^{n}\left\{ \frac{1}{M_{i}} \sum_{j=1}^{M_{i}} \hat{\phi}_{i j l}^{F}(t ; \boldsymbol{z}_{0})\right\}\left\{ \frac{1}{M_{i}} \sum_{j=1}^{M_{i}} \hat{\phi}_{i j l}^{F}(s; \boldsymbol{z}_{0})\right\}$, $t, s \in[0, \tau]$. Similar to the case of the cumulative baseline hazards $\Lambda_{0,l}$, a $1-\alpha$ confidence band for $F_{0,l}(\cdot ; \boldsymbol{z}_{0})$ can be constructed as $g^{-1} \left[g\left\{\hat{F}_{0,l}(t ; \boldsymbol{z}_{0})\right\} \pm \frac{c_{\alpha}}{\sqrt{n} q_{l}^{F}\left(t ; \boldsymbol{z}_{0}\right)}\right]$, $t \in [t_{1}, t_{2}]$, where $c_{\alpha}$ is the $1-\alpha$ quantile of the distribution of $\sup _{t \in[t_{1}, t_{2}]} |\hat{B}_{n, l}^{F}(t ; \boldsymbol{z}_{0})|$, with $\hat{B}_{n, l}^{F}(t ; \boldsymbol{z}_{0})=q_{l}^{F}(t ; \boldsymbol{z}_{0}) \dot{g}\{\hat{F}_{n, l}(t ; \boldsymbol{z}_{0})\} \hat{W}_{n, l}^{F}(t ; \boldsymbol{z}_{0})$. A standard transformation to ensure that the limits of the bands for the cumulative incidence functions reside in $[0,1]$ is $g(x)=\log \{-\log (x)\}$ (Cheng *and others,* 1998). The weight function choice $q_{l}^{F}(t ; \boldsymbol{z}_{0})=\hat{F}_{n,l}(t ; \boldsymbol{z}_{0}) \log \{\hat{F}_{n,l}(t ; \boldsymbol{z}_{0})\}\{\hat{\sigma}_{F_{l}}(t ; \boldsymbol{z}_{0})\}^{-1}$, with $\hat{\sigma}_{F_{l}}\left(t ; \boldsymbol{z}_{0}\right)$ being the square root of the estimated variance of $\hat{F}_{0,l}(t ; \boldsymbol{z}_{0})$, provides an equal-precision-type band; the choice $q_{l}^{F}(t ; \boldsymbol{z}_{0})=\hat{F}_{n,l}(t ; \boldsymbol{z}_{0}) \log \{\hat{F}_{n,l}(t ; \boldsymbol{z}_{0})\}\{1+\hat{\sigma}^{2}_{F_{l}} (t ; \boldsymbol{z}_{0})\}^{-1}$, provides a Hall–Wellner-type band.

## 3. Simulation studies

In our simulation studies, we generated data from a hypothetical multicenter study with two competing risks, where cause 1 was the event of primary scientific interest. To mimic a realistic setting, we induced a moderate positive association across individuals from the same cluster/study center, introduced ICS, and considered missing values in a cause of failure, with the probability of missingness depending on an auxiliary variable. To evaluate the appropriateness of our methods for data analysis in such a realistic study setting, we assessed the biases of (i) the estimated regression coefficients, (ii) the baseline cumulative hazard estimates, (iii) the cumulative incidence function estimates, and the (iv) estimated standard errors. We also evaluated the appropriateness of our 9$\%$ CIs and confidence bands for the parameters of interest in the above setting. The specifics of the simulation studies are described below.

We considered two covariates $\boldsymbol{Z}=(Z_{1}, Z_{2})^{T}$, where $Z_1 \sim N(0,2^2)$ and $Z_2\sim {\rm Bernoulli}(0.5)$, and with the observation time interval being $[0,2]$. The right censoring times were independently generated from the ${\rm Exp}(0.4)$ distribution. For each cause, the failure times were generated from Cox proportional hazards shared frailty models with a positive stable frailty (Hougaard, 1986; Cong *and others,* 2007; Liu *and others,* 2011) to introduce within-cluster dependence. These models had the form $\lambda_{l}(t \mid Z_{ij,l},w_{i,l})=\lambda_{0,l}(t)w_{i,l}\exp(\beta_{0,l} Z_{ij,l})$, $l=1,2$, where $w_{i,l}$ followed a positive stable distribution with parameter $\alpha=0.5$, which induced a moderate within-cluster dependence. This type of dependence is expected to be similar to that in our motivating EA-IeDEA study. This simulation setup leads to the marginal cause-specific hazard functions $\lambda_{l}(t \mid {Z}_{ij,l})=\alpha \lambda_{0,l}(t)\Lambda_{0,l}(t)^{\alpha-1}\exp(\alpha{\beta}_{0,l}{Z}_{ij,l})$, $l=1,2$, which are still proportional, owing to the positive stable frailty, with true parameters ${\beta}^{\prime}_{0,l}=\alpha{\beta}_{0,l}$, and $\Lambda_{0,l}^{\prime}(t)={\Lambda_{0,l}(t)}^{\alpha}$.

In this simulation study, we considered two scenarios, with settings similar to those used in the simulation studies of Hyun *and others* (2012) and Bakoyannis *and others* (2020). In both scenarios, the event time for the cause $1$ was generated assuming $\lambda_{0,1}(t)=1$ and $\beta_{0,1}=-0.5$. In Scenario 1, the event time for cause $2$ was simulated from a Gompertz distribution with baseline hazard $\lambda_{0,1}(t)=\exp(-0.5+0.2t)$, and $\beta_{0,2}=-0.5$. In Scenario 2, the event time for cause $2$ was simulated from a Weibull distribution with baseline hazard $\lambda_{0,1}(t)=\{2\sqrt{2t}\}^{-1}$ and $\beta_{0,2}=-0.5$. The simulation setup under Scenario 1 led to on average in $13.5\%$ right-censored observations, $50.4\%$ failures from cause 1, and $36.1\%$ failures from cause 2. The corresponding figures for Scenario 2 were $12.7\%$, $41.8\%$, and $45.5\%$. The implied model for $\pi_1(\boldsymbol{W}_{i j},\boldsymbol{\gamma}_0)$ had approximately linear time effect with the form ${\rm logit}\{\pi_1(\boldsymbol{W}_{i j},\boldsymbol{\gamma}_0)\}\approx\gamma_{0}+\gamma_{1}T+\gamma_{2}Z_{1}+\gamma_{3}Z_{2}$ under Scenario 1, where $\boldsymbol{\gamma}_0\approx(0.25,-0.15,-0.25,0.25)^T$, and had the form ${\rm logit}\{\pi_1(\boldsymbol{W}_{i j},\boldsymbol{\gamma}_0)\}=\gamma_{0}+\gamma_{1}\log(T)+\gamma_{2}Z_{1}+\gamma_{3}Z_{2}$ under Scenario 2, where $\boldsymbol{\gamma}_0=(5\log(2)/4,0.25,-0.25,0.25)^T$. The missingness indicators $R_{ij}$ were generated under the logistic model ${\rm logit}\left\{P(R_{ij}=1\mid\Delta_{ij}=1,\boldsymbol{W}_{ij})\right\}=(1,\boldsymbol{W}^{T}_{ij})\boldsymbol{\theta}$, where $\boldsymbol{W}^{T}=(T,Z_{1},Z_{2})$. We considered the parameter values $\boldsymbol{\theta}=(0.7,1,-1,1)^{T}$, $(-0.2,1,-1,1)^{T}$, and $(-0.8,1,-1,1)^{T}$, which resulted in $24.5\%$, $35.1\%$, and $42.8\%$ missing causes of failure in Scenario 1, and $25.5\%$, $36.3\%$, and $44.1\%$ missingness in Scenario 2.

In this simulation study, we considered $n=50$, $100$, and $200$ which correspond to situations with small to moderate number of clusters. To introduce ICS, the cluster sizes $M_i$ were generated from a mixture of discrete uniform distributions depending on the frailty, with $M_i \sim {\rm Unif}(20,30)$ if $w_{i,1} \lt {\rm median}(w_1)$ and $w_{i,2} \lt {\rm median}(w_2)$, $M_i \sim {\rm Unif}(50,60)$ if $w_{i,1}\ge {\rm median}(w_1)$ and $w_{i,2}\ge{\rm median}(w_2)$, and $M_i \sim {\rm Unif}(30,50)$, otherwise. For each simulation setting, we simulated 1000 data sets, and analyzed each data set using the proposed method and the method by Bakoyannis *and others* (2020). All analysis assumed the parametric model ${\rm logit}\{\pi_1(\boldsymbol{W}_{i j},\boldsymbol{\gamma}_0)\}=\gamma_{0}+\gamma_{1}T+\gamma_{2}Z_{1}+\gamma_{3}Z_{2}$, which was approximately correctly specified under Scenario 1, and misspecified under Scenario 2. The standard errors were estimated using the closed-form formulas provided in Section 2. The $95\%$ confidence bands for $\Lambda_{0,1}(t)$ and $F_{0,1}(t)$ were computed based on 1000 simulation realizations standard normal variables $\left\{\xi_{i}\right\}_{i=1}^{n}$ as described in Section 2. The limits of the time domain $[t_1,t_2]$ for the confidence bands were chosen to be $10\%$ and $90\%$ percentile of the observed failure times.

The simulation results for the regression coefficient $\beta_{1}$ under Scenario 1 are summarized in Table 1. The proposed estimator was approximately unbiased and the average standard errors were close to the Monte Carlo standard deviations. This provides numerical evidence for the consistency of our estimator of the regression coefficient $\hat{\beta}_{n,1}$ and its associated standard error. The $95\%$ coverage probabilities were close to the nominal level in all cases. In contrast, the method by Bakoyannis *and others* (2020) provided biased estimates. The bias for $\beta_{1}$ was relatively small under Scenario 1, with an increasing trend as the number of clusters increased. The average standard errors were smaller than the Monte Carlo standard deviation, which implies that the standard errors were underestimated. This resulted in poor coverage probabilities of the corresponding $95\%$ CIs. The poor performance of the method by Bakoyannis *and others* (2020) is to be expected as this method is not intended to be applied to clustered data or to address the ICS issue.

**Table 1.** Simulation results for the regression coefficient $\beta_{1}$ under Scenario 1 for the proposed approach and the approach by Bakoyannis *and others* (2020) (BZY20) which ignores the within-cluster dependence

		Proposed				BZY20			
$n$	$p_m(\%)$	Bias	MCSD	ASE	CP	Bias	MCSD	ASE	CP
50	25	$-$ 0.006	0.033	0.032	0.937	0.003	0.033	0.021	0.782
	35	$-$ 0.006	0.034	0.033	0.938	0.003	0.035	0.023	0.793
	43	$-$ 0.006	0.036	0.034	0.939	0.004	0.035	0.025	0.827
100	25	$-$ 0.002	0.022	0.022	0.949	0.007	0.022	0.015	0.777
	35	$-$ 0.002	0.023	0.023	0.941	0.007	0.023	0.016	0.803
	43	$-$ 0.002	0.024	0.024	0.948	0.007	0.024	0.018	0.822
200	25	$-$ 0.001	0.016	0.016	0.954	0.008	0.016	0.010	0.735
	35	$-$ 0.001	0.017	0.017	0.953	0.008	0.017	0.011	0.762
	43	$-$ 0.001	0.017	0.017	0.953	0.009	0.017	0.013	0.785

*n*,: number of clusters with cluster size $M \in [30,60]$; $p_m$, percentage of missingness; MCSD, Monte Carlo standard deviation; ASE, average estimated standard error; CP, coverage probability of $95\%$ confidence interval.

The simulation results for the pointwise estimates of the infinite-dimensional parameters $\Lambda_{0,1}(t)$ and $F_{0,1}(t)$ under Scenario 1 are provided in Supplementary material available at *Biostatistics* online. Our proposed estimators had good performance with small bias, average standard errors close to the Monte Carlo standard deviation, and $95\%$ CI coverage probabilities close to the nominal level. As expected, the method by Bakoyannis *and others* (2020) provided estimators with large bias, severely under-estimated standard errors, and poor coverage probabilities of the $95\%$ CIs. Table 2 presents the coverage probabilities of $95\%$ simultaneous confidence bands for $\Lambda_{0,1}(\cdot)$ and $F_{0,1}(\cdot)$ under Scenario 1. The proposed $95\%$ confidence bands had coverage probabilities close to the 0.95 level, which indicates the appropriateness of our bands with small numbers $n$ of clusters. As expected, the coverage probabilities of the $95\%$ confidence bands by Bakoyannis *and others* (2020) were considerably lower than the 0.95 level. This is expected to reflect the under-estimation of the standard error, which leads to narrower bands, and the bias in the point estimates, which is attributed to the ICS.

**Table 2.** Simulation results for the coverage probabilities of $95\%$ simultaneous confidence bands for the infinite-dimensional parameters $\Lambda_{0,1}(t)$ and $F_{0,1}(t)$ under Scenario 1. Results from the proposed approach and the approach by Bakoyannis *and others* (2020) (BZY20) which ignores the within-cluster dependence

		$\Lambda_{0,1}(t)$				$F_{0,1}(t)$			
$n$	$p_m(\%)$	Proposed		BZY20		Proposed		BZY20	
		EP	HW	EP	HW	EP	HW	EP	HW
50	25	0.900	0.936	0.077	0.153	0.906	0.931	0.120	0.203
	35	0.912	0.936	0.105	0.185	0.904	0.928	0.146	0.245
	43	0.914	0.939	0.130	0.213	0.911	0.929	0.167	0.268
100	25	0.931	0.945	0.049	0.092	0.931	0.952	0.096	0.150
	35	0.931	0.947	0.064	0.114	0.932	0.951	0.106	0.173
	43	0.937	0.948	0.082	0.132	0.939	0.953	0.141	0.210
200	25	0.942	0.947	0.016	0.034	0.938	0.955	0.073	0.108
	35	0.940	0.950	0.025	0.046	0.945	0.954	0.085	0.129
	43	0.942	0.951	0.041	0.062	0.945	0.955	0.105	0.154

$n$ , number of clusters with cluster size $M \in [30,60]$; $p_m$, percentage of missingness; EP, equal precision bands; HW, Hall–Wellner-type bands.

The simulation results under Scenario 2, where the assumed model $\pi_1(\boldsymbol{W},\boldsymbol{\gamma}_0)$ was misspecified, are provided in the Supplementary material available at *Biostatistics* online. The results for point estimates under this scenario were similar to those form Scenario 1 (Table 1). This provides numerical evidence for the robustness of the proposed approach under some degree of model misspecification in $\pi_l(\boldsymbol{W},\boldsymbol{\gamma}_0)$. However, the confidence bands had lower coverage rates under Scenario 2. This illustrates the importance to evaluate the goodness of fit of $\pi_l(\boldsymbol{W},\boldsymbol{\gamma}_0)$ in practice, as described in Section 2.3.

## 4. HIV data application

The proposed method was applied to the electronic health record data from the EA-IeDEA study to identify risk factors for disengagement from HIV care and death while in care (i.e., prior to a disengagement). Disengagement from care and death while in care were the two competing risks of interest. Disengagement from care was defined by the clinical investigators of the study as being alive and without HIV care for 2 months. The covariates of interest were sex, age, CD4 count at ART initiation, and HIV status disclosure. The data set included 24 372 HIV-infected adult patients from 30 clinics who initiated ART on/after January 1, 2010. The median (first quartile, third quartile) number of patients in a clinic (cluster size) was 465 (209, 1049). Throughout the follow-up period, 8082 patients remained in care (right censoring), 84 died while in care (reported death), and 16 206 were lost to clinic for at least 2 months. Among the 1206 lost patients, 5107 (31.5$\%$) were intensively outreached in the community and their vital status was actively ascertained by outreach workers. Of these patients, 1867 (36.6$\%$) were found to be deceased within 2 months from the last clinic visit, which indicates a substantial death under-reporting issue. The remaining 11 099 lost patients who were not outreached had a missing cause of failure. Descriptive statistics for the study sample are presented in Table S5 of the Supplementary material available at *Biostatistics* online.

In the first step of the analysis, we fitted a model for the probability of death among those who were lost to the clinic (i.e., unreported death). The model had the form

\begin{equation*} {\rm logit}\{\pi_1(\boldsymbol{W},\boldsymbol{\gamma}_0)\} = \gamma_{0}+\gamma_{1}T+\gamma_{2} I({Sex} =``male'')+\gamma_{3}Age + \gamma_{4}CD4 + \gamma_{5}I(HIVstatus=``yes''),\end{equation*}

where $T$ is the time from ART initiation to any event and $HIVstatus$ corresponds to HIV status disclosure. The estimated residual process for the latter model (depicted in Figure S1 of the Supplementary material available at *Biostatistics* online) fell within the 95$\%$ simultaneous confidence band under the null hypothesis of a good model fit ($p$-value = 0.850). This indicates that there is no evidence for a violation of the parametric model assumption imposed for this data set. The parameter estimates $\hat{\boldsymbol{\gamma}}_n$ for this model along with the associated standard errors and $p$-values are presented in Table 3. These results indicate that males and older patients had an increased odds of an unreported death. In contrast, the odds of death under-reporting were lower for those with a higher CD4 cell count and patients who remained in care for a longer period of time. These results are expected to reflect the fact that younger, healthier (according to the CD4 cell count), and more compliant patients are less likely to die.

**Table 3.** First step of the data analysis for the EA-IeDEA study. Analysis results for the probability of death among those lost to clinic (i.e., probability of an unreported death) $\pi_1(\boldsymbol{W},\boldsymbol{\gamma}_0)$ based on a marginal logit model

Covariates	$\hat{\gamma}_n$	SE	*p*-value	OR (95$\%$ CI)
(Intercept)	–0.379	0.185	0.040	—
Time since ART initiation (per year)	–0.454	0.057	<0.001	0.635 (0.568, 0.711)
Sex (male = 1, female = 0)	0.303	0.120	0.011	1.353 (1.071, 1.711)
Age (per 10 years)	0.181	0.067	0.007	1.199 (1.051, 1.367)
CD4 (per 100 cells/$\mu$l)	–0.488	0.051	<0.001	0.614 (0.555, 0.678)
HIV status (Yes = 1, No = 0)	0.353	0.209	0.091	1.423 (0.945, 2.144)

OR, marginal odds ratio; 95$\%$ CI, 95$\%$ confidence interval; HIV status, HIV status disclosed.

In the second step of the analysis (main analysis), we fitted the marginal proportional cause-specific hazard models of interest

\begin{equation*} \lambda_{l}\left(t ; \boldsymbol{Z}\right)=\lambda_{0,l}(t) \exp\left\{\beta_{l,1}I(Sex=``male'') + \beta_{l,2} Age + \beta_{l,3} CD4 + \beta_{l,4} I(HIVstatus=``yes'')\right\},\end{equation*}

$l=1,2$, using the proposed approach along with the parameter estimates $\hat{\boldsymbol{\gamma}}_n$ from the first step. For comparison purposes, we also used the method by Bakoyannis *and others* (2020). The results from this analysis are presented in Table 4. The results from the proposed method show that younger patients and those with a higher CD4 count at ART initiation had a higher hazard of disengagement from HIV care. This can be explained by the fact that younger and healthier individuals are expected to have a lower motivation for engaging in care. Males and patients with a lower CD4 count (and thus with a more advanced HIV disease) at ART initiation had a higher hazard of death while in care. Unlike the analysis based on the proposed approach, the method by Bakoyannis *and others* (2020) which ignores the within-cluster dependence and the ICS, provided significant sex and HIV status disclosure effects on the hazard of disengagement from HIV care, and significant age and HIV status disclosure effects on the hazard of death while in care. The dubiously significant effects from the naïve analysis may be attributed to the under-estimation of standard errors, in addition to the bias due to the potential ICS.

**Table 4.** Second step of the data analysis for the EA-IeDEA study. Results from the proposed approach and the approach by Bakoyannis *and others* (2020) (BZY20) which ignores the within-cluster dependence

	Proposed$^\dagger$			BZY20^‡^		
Covariates	$\exp(\hat{\beta}_n)$	95$\%$ CI$^\S$	*p*-value	$\exp(\hat{\beta}_n)$	95$\%$ CI$^\S$	*p*-value
Disengagement from HIV care						
Sex (male = 1, female = 0)	0.97	(0.85, 1.12)	0.720	1.07	(1.01, 1.12)	0.016
Age (per 10 years)	0.78	(0.73, 0.83)	<0.001	0.77	(0.75, 0.79)	<0.001
CD4 (per 100 cells/$\mu$l)	1.07	(1.01, 1.13)	0.021	1.05	(1.04, 1.06)	<0.001
HIV status^¶^ (Yes = 1, No = 0)	0.94	(0.82, 1.07)	0.350	0.83	(0.79, 0.87)	<0.001
Death while in care						
Sex (male = 1, female = 0)	1.35	(1.12, 1.62)	<0.001	1.30	(1.19, 1.41)	<0.001
Age (per 10 years)	1.00	(0.89, 1.11)	1.000	1.07	(1.03, 1.12)	0.001
CD4 (per 100 cells/$\mu$l)	0.63	(0.57, 0.69)	<0.001	0.67	(0.64, 0.71)	<0.001
HIV status^¶^ (Yes = 1, No = 0)	1.17	(0.89, 1.53)	0.250	1.27	(1.16, 1.39)	<0.001

$^\dagger$ The standard errors were estimated with cluster bootstrap.

^‡^The standard errors were estimated with bootstrap at individual level.

$^\S$ 95$\%$ confidence interval for the cause-specific hazard ratio $\exp(\beta_0)$.

^¶^HIV status disclosed.

**Fig. 1.**
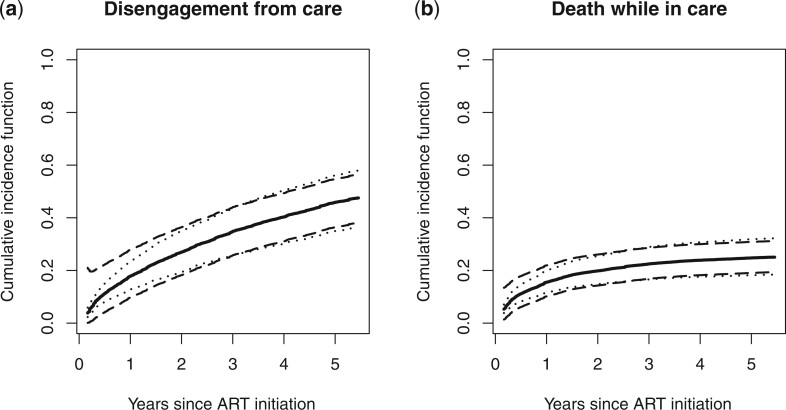
Plot for predicted cumulative incidence functions (solid lines) of (a) disengagement from HIV care and (b) death while in care with the $95\%$ simultaneous confidence bands based on equal precision bands (dotted lines) and Hall–Wellner bands (dashed lines), for a 40-year-old male with a CD4 count of 150 cells/$\mu$l at ART initiation and undisclosed HIV status.

To illustrate the proposed method for risk prediction, we depicted the predicted cumulative incidence functions of (i) disengagement from care and (ii) death while in care for a 40-year-old male with a CD4 count of 150 cells/$\mu$l at ART initiation and undisclosed HIV status, along with the 95$\%$ equal precision and Hall–Wellner-type bands in Figure 1. The bands reflect the accuracy of the estimated cumulative incidence functions based on the EA-IeDEA data. The predicted cumulative incidence (equal precision 95$\%$ confidence limits; Hall–Wellner-type 95$\%$ confidence limits) of disengagement from care for such patient at 1, 2, and 5 years since ART initiation were 0.178 (0.128–0.235; 0.097–0.278), 0.269 (0.194–0.349; 0.182–0.364), and 0.459 (0.350–0.561; 0.365–0.548), respectively. The corresponding figures for the cumulative incidence of death while in care were 0.155 (0.116–0.200; 0.101–0.219), 0.198 (0.148–0.254; 0.143–0.260), and 0.248 (0.182–0.312; 0.191–0.309). These results indicate that a 40-year-old male patient with a CD4 count of 150 cells/$\mu$l at ART initiation and undisclosed HIV status is quite likely to disengage from care by 5 years since ART initiation. Therefore, more intensive health education to increase this patient’s motivation to engage in care is needed. In addition, such a patient’s mortality risk is quite high which may warrant more intensive care.

## 5. Discussion

In this article, we proposed a general framework for marginal semiparametric regression analysis of clustered competing risks data with missing cause of failure. Our methodology utilizes the marginal proportional cause-specific hazards model and uses a two-step partial pseudolikelihood approach for estimation under a MAR assumption. We provide estimators for both regression coefficients and infinite-dimensional parameters, such as the marginal cumulative incidence function. The proposed method does not impose assumptions regarding the within-cluster dependence and also accounts for ICS. The proposed estimators were shown to be strongly consistent and asymptotically normal. Closed-form variance estimators were provided and a rigorous methodology for the calculation of simultaneous confidence bands for the infinite-dimensional parameters was proposed. Our simulation studies showed that the performance of the method was satisfactory even with a very small number of clusters and, also, under a misspecified parametric model for the cause of failure $\pi_{1}(W,\boldsymbol{\gamma}_0)$. As expected, the previously proposed method by Bakoyannis *and others* (2020), which is not intended to be applied to clustered data or to address the ICS issue, resulted in biased estimates, underestimated standard errors, and poor coverage probabilities. The analysis of the EA-IeDEA data illustrated that ignoring the within-cluster dependence and the ICS may lead to dubiously significant results in practice. Last but not least, our proposed method can be easily implemented using the R code presented in the Supplementary material available at *Biostatistics* online. This code was used in our simulation studies and HIV data analysis. Even though the proposed methodology provides inference for the TCM population, it can be modified to provide inference for the ACM population by simply removing the weight $1/M_i$ from the partial pseudoscore function $\boldsymbol{G}_{n,l}\left(\boldsymbol{\beta}; \hat{\boldsymbol{\gamma}}_n\right)$, the estimators $\hat{\Lambda}_{n,l}(t)$, $l=1,\ldots,k$, and the empirical influence functions.

To the best of our knowledge, the issue of clustered competing risks data with the missing cause of failure has only been addressed using frailty models by Lee *and others* (2017). However, this methodology imposes strong assumptions regarding the within-cluster dependence and the distribution of the random effects, which may be violated in practice. Moreover, this approach does not account for ICS and does not provide inference about the infinite-dimensional parameters such as the cumulative incidence function. Nevertheless, the covariate-specific cumulative incidence functions are essential for personalized risk prediction in modern medicine. Finally, the method is computationally intensive and provides cluster-specific inference, even though the population-averaged inference is more scientifically relevant in many applications including our motivating EA-IeDEA study. The methodology presented in this paper effectively addresses all these limitations and is the first, to the best of our knowledge, rigorous approach for marginal analysis of clustered competing risks data with ICS and missing causes of failure.

A key assumption of our methodology is the MAR assumption. This assumption can be made more plausible in applications by incorporating auxiliary variables that may be related to the probability of missingness (Lu and Tsiatis, 2001; Nevo *and others,* 2018). In addition, our method adopted a parametric model for the marginal cause of failure probability $\pi_l(\boldsymbol{W},\boldsymbol{\gamma}_{0})$, $l=1,\ldots,k$. Our simulation studies provided numerical evidence that our regression parameter estimators are robust against some degree of model misspecification. However, the confidence bands had a lower coverage rate under a misspecified model for the cause of failure probability. To this end, we suggest the use of the goodness-of-fit procedure based on the cumulative residual process to evaluate the model assumption for $\pi_l(\boldsymbol{W},\boldsymbol{\gamma}_{0})$, $l=1,\ldots,k$, in practice. In our HIV data application, the use of this approach revealed that there was no evidence for a violation of the parametric model assumption. In applications where there is evidence for lack of fit, we suggest the use of more flexible parametric models including regression splines to alleviate the impact of misspecification.

There are many possible extensions to this work. One may relax the MAR assumption by utilizing instrumental variables (Tchetgen Tchetgen and Wirth, 2017). Moreover, considering nonparametric or semiparametric models for $P(C_{i j}=l|\Delta_{i j}=1,\boldsymbol{W}_{i j})$, or machine learning methods to predict the missing causes of failure is of great practical and methodological interest.

## Supplementary Material

kxac012_Supplementary_Data

## 6. Software

Software in the form of an R package is available at https://github.com/wz11/ClusteredMPPLE.
