# Supplementary material for: Semiparametric marginal regression for clustered competing risks data with missing cause of failure
Source: Biostatistics. 2022 Apr 12;24(3):795–810. doi: 10.1093/biostatistics/kxac012 (PMC10345995; doi:10.1093/biostatistics/kxac012)
Supplement: kxac012_Supplementary_Data [file kxac012_supplementary_data.pdf]

Biostatistics (2021), **0**, 0, *pp.* 1–26
doi:10.1093/biostatistics/Clust_MPPLE_Supp_R1

Supplementary Material for 'Semiparametric Marginal Regression Analysis for Clustered Competing Risks Data with Missing Cause of Failure'

Wenxian Zhou[1], Giorgos Bakoyannis[1,*], Ying Zhang[2],

Constantin T. Yiannoutsos[1]

[1] *Department of Biostatistics, Indiana University, Indianapolis, Indiana, U.S.A.*

[2] *Department of Biostatistics, University of Nebraska Medical Center, Omaha, Nebraska, U.S.A.*

*gbakogia@iu.edu

1. R Code

Our methodology can be easily implemented using standard software that allows for weights. In this Appendix we illustrate the use of the `coxph` function in the R package `survival`. Let `data` be a data set with the variables: cluster id `clusterid`, observed time `x`, cause of failure `c`, missingness indicator `r`, a covariate of interest `z`, an auxiliary variable `a`, and cluster size `clustersize`.

For illustration purposes, we analyze the cause specific hazard for cause 1 here. The first stage of the analysis is to estimate γ_0 through generalized estimating equations (GEE) for logistic regression using the observations with an observed cause of failure. This can be done using the following code.

```
cause <- 1
data$include <- 1*(data$r==1 & data$c>0)/data$clustersize
```

© The Author 2021. Published by Oxford University Press. All rights reserved. For permissions, please e-mail: journals.permissions@oup.com

```
data$outcome <- 1*(data$c==cause)
model <- geeglm( outcome ~ x + z + a, family = "binomial",
    data = data, id = clusterid, weight = include, corstr = "independence")
```

Then, one needs to calculate the estimated probability of the cause of failure $\pi_1(\boldsymbol{W}_{ij}, \hat{\gamma}_n)$.

```
data$yhat <- predict(model, data, type = "response")
```

The second stage of the analysis is to maximize the weighted partial pseudolikelihood. This can be implemented with the `coxph` function using the weight option and some simple data management. The data management steps are used to "remove" the weights from the risk sets for the observations with a missing cause of failure.

```
data$d <- data$r*(data$c == cause) + (1-data$r)*(data$yhat > 0)
data$weight <- data$r + (1-data$r)*data$yhat
data$weight <- data$weight + (data$weight == 0)
dt0 <- data[data$r==0, ]
dt0$weight <- 1 - dt0$weight
dt0$d <- 0
data1 <- rbind(data, dt0)
data1$weight <- data1$weight/data1$clustersize
```

Then the point estimates of the regression coefficient can be calculated using the augmented dataset `data1` as follows.

```
mod <- coxph(Surv(x, d) ~ z, weight = weight, data = data1)
beta1 <- coef(mod)
```

The cause-specific baseline cumulative hazard function can be calculated with the `basehaz` function as follows.

```
H1 <- basehaz(mod, centered = FALSE)
```

Finally, we can get the estimated baseline cumulative incidence function using equation (1) in the main manuscript, based on the estimated regression coefficients and baseline cumulative hazard functions for all causes of failure. For example, in the case of two causes of failure, the baseline cumulative incidence function can be estimated as follows.

```
Haz1 <- H1$hazard
Haz2 <- H2$hazard
S <- exp(- Haz1 - Haz2)
S.minus <- c(1, S[1: (length(S) - 1)])
Haz1.minus <- c(0, Haz1[1: (length(Haz1) - 1)])
CIF1 <- cumsum(S.minus * (Haz1 - Haz1.minus))
```

The computation of our closed-form standard error estimators is more involved. However, these standard error estimators are readily available in our R functions that can be found at `https://github.com/wz11/ClusteredMPPLE`. We must note that the uncertainty in the estimate $\hat{\gamma}_n$ that appears in the weights is properly incorporated into our standard error estimators. This is due to the fact that our standard error estimators involve appropriately the empirical versions of the influence functions $\boldsymbol{\omega}_{ij}$ for $\hat{\gamma}_n$ (see Section 2.3 of the main manuscript and regularity condition C4 in Section 2 of this Supplementary Material). Alternatively, one can use the nonparametric cluster bootstrap approach for standard error estimation (Bakoyannis, 2021).

2. Asymptotic Theory Proofs

The asymptotic properties of the proposed estimators are justified based on empirical process theory (Kosorok, 2008; van der Vaart and Wellner, 1996). We use the following standard empirical

process notations throughout the Appendix B. For any measurable functions $f : \mathcal{D} \mapsto \mathbb{R}$,

$$\mathbb{P}_n f = \frac{1}{n}\sum_{i=1}^{n} f(\boldsymbol{D}_i), \text{ and } Pf = \int_{\mathcal{D}} f dP = Ef(\boldsymbol{D}_1).$$

Also, let K_0 denote a generic constant that may change from place to place. For simplicity, we will omit the subindex i, indicating a specific cluster, from expectations. By the i.i.d. assumption across clusters, the expectations correspond to expectations of (functions of) random variables from an arbitrary cluster. In the proofs we will consider an arbitrary cause of failure $l \in \{1, \dots, k\}$. Before stating the regularity conditions, we define the negative of the Hessian matrix of the expected partial pseudolikelihood as

$$\boldsymbol{H}_l\left(\boldsymbol{\beta}\right) = E\left\{\frac{1}{M}\sum_{j=1}^{M}\int_0^{\tau} \boldsymbol{V}\left(t, \boldsymbol{\beta}\right) d\tilde{N}_{jl}\left(t; \boldsymbol{\gamma}_0\right)\right\},$$

where

$$\boldsymbol{V}(t,\boldsymbol{\beta}) = \frac{E\{\frac{1}{M}\sum_{j=1}^{M} Y_j(t)\exp(\boldsymbol{\beta}^T\boldsymbol{Z}_j)\boldsymbol{Z}_j^{\otimes 2}\}}{E\{\frac{1}{M}\sum_{j=1}^{M} Y_j(t)\exp(\boldsymbol{\beta}^T\boldsymbol{Z}_j)\}} - \left[\frac{E\{\frac{1}{M}\sum_{j=1}^{M} Y_j(t)\exp(\boldsymbol{\beta}^T\boldsymbol{Z}_j)\boldsymbol{Z}_j\}}{E\{\frac{1}{M}\sum_{j=1}^{M} Y_j(t)\exp(\boldsymbol{\beta}^T\boldsymbol{Z}_j)\}}\right]^{\otimes 2}.$$

In this work we assume the following regularity conditions.

C1. $\Lambda_{0,l}(t)$ is a non-decreasing continuous function with $\Lambda_{0,l}(\tau) < \infty$, for $l = 1, \dots, k$ and $E\{Y(\tau) \mid \boldsymbol{Z}, M\} > 0$ almost surely.

C2. The true regression coefficients $\boldsymbol{\beta}_{0,l} \in \mathcal{B}_l \subset \mathbb{R}^{p_l}$, where $\mathcal{B}_l$ is bounded and convex set for $l = 1, \dots, k$ and $\boldsymbol{\beta}_{0,l}$ is in interior of $\mathcal{B}_l$.

C3. The inverse of the link function g for the marginal probability model of the cause of failure $\pi_l(\boldsymbol{W}_{ij}, \boldsymbol{\gamma}_0)$, $l = 1, \dots, k$, has continuous derivative $\dot{g}$ with respect to $\boldsymbol{\gamma}_0$ on compact sets. The parameter space Γ of $\boldsymbol{\gamma}_0$ is a bounded subset of $\mathbb{R}^{p_\gamma}$.

C4. The estimating function for the model of the cause of failure is Lipschitz continuous in $\boldsymbol{\gamma}$, and the estimator $\hat{\boldsymbol{\gamma}}_n$ is strongly consistent and asymptotically linear, i.e. $\sqrt{n}(\hat{\boldsymbol{\gamma}}_n - \boldsymbol{\gamma}_0) =$

$\frac{1}{\sqrt{n}}\sum_{i=1}^{n}\frac{1}{M_i}\sum_{j=1}^{M_i}\boldsymbol{\omega}_{ij}+o_p(1)$, where $\boldsymbol{\omega}_{ij}$ is the influence function of jth subject in ith cluster, satisfying $E(\boldsymbol{\omega}_{ij})=0$ and $E\left\|\boldsymbol{\omega}_{ij}\right\|^2<\infty$.

C5. The covariates of interest $\boldsymbol{Z}$, the auxiliary covariates $\boldsymbol{A}$, and the cluster size M are bounded, in the sense that there exist constants $K\in\mathbb{R}_+$ and $m_0\in\mathbb{N}_+$ such that $P(\|\boldsymbol{Z}\|\vee\|\boldsymbol{A}\|\leqslant K)=1$ and $P(M\leqslant m_0)=1$.

C6. The Hessian matrix of the expected partial pseudolikelihood $-\boldsymbol{H}_l(\boldsymbol{\beta})$ is negative definite on $\mathcal{B}_l$ for all $l=1,\dots,k$.

C7. The observations W_{ij}, Δ_{ij}, ϵ_{ij}, and R_{ij} are identically distributed conditionally on cluster size M_i, in the sense that $E(\boldsymbol{W}_{ij}|M_i)=E(\boldsymbol{W}_{i1}|M_i)$, $E(\Delta_{ij}|M_i)=E(\Delta_{i1}|M_i)$, $E(\epsilon_{ij}|M_i\}=E(\epsilon_{i1}|M_i)$, and $E(R_{ij}|M_i)=E(R_{i1}|M_i)$, for all $i=1,\dots,n$, $j=1,\dots,M_i$, and $l=1,\dots,k$.

Regularity conditions C3 and C4 are satisfied when the marginal model for $\pi_l(\boldsymbol{W}_{ij},\boldsymbol{\gamma}_0)$ is correctly specified with a standard link function, such as the logit link, and parameters estimated through generalized estimating equations under a working independence assumption. The assumptions on the parametric models for $\pi_l(\boldsymbol{W}_{ij},\boldsymbol{\gamma}_0)$ can be evaluated using the cumulative residual processes $E\left[\frac{1}{M}\sum_{j=1}^{M}R_j\{N_{jl}(t)-\pi_l(\boldsymbol{W}_j,\boldsymbol{\gamma}_0)N_j(t)\}\right]$, $l=1,\dots,k-1$, $t\in[0,\tau]$, which can be estimated by $\frac{1}{n}\sum_{i=1}^{n}\frac{1}{M_i}\sum_{j=1}^{M_i}R_{ij}\{N_{ijl}(t)-\pi_l(\boldsymbol{W}_{ij},\hat{\boldsymbol{\gamma}}_n)N_{ij}(t)\}$. If the model is correctly specified, the cumulative residual process is equal to 0 for $t\in[0,\tau]$. A formal goodness of fit test can be conducted using the simulation-based approach by Pan and Lin (2005). A graphical evaluation of goodness of fit can also be performed by plotting the observed residual process and the 95% simultaneous confidence band around the line $f(t)=0$, $t\in[0,\tau]$ (Bakoyannis *and others*, 2019, 2020).

Before presenting the proofs of the theorems stated in the main manuscript, we provide explicit formulas for the elements of the influence functions. First, for Theorem 2.2, define

$$\boldsymbol{\psi}_{ijl}=\boldsymbol{H}_l^{-1}(\boldsymbol{\beta}_{0,l})\int_0^{\tau}\{\boldsymbol{Z}_{ij}-\boldsymbol{E}(t,\boldsymbol{\beta}_{0,l})\}d\tilde{M}_{ijl}(t;\boldsymbol{\beta}_{0,l},\boldsymbol{\gamma}_0),$$

where $\boldsymbol{H}_l(\boldsymbol{\beta}_{0,l})$ is the negative of the Hessian matrix of the expected partial pseudolikelihood

and

$$\boldsymbol{E}(t,\boldsymbol{\beta}_{0,l}) = \frac{E\{\frac{1}{M}\sum_{j=1}^{M} Y_j(t)\exp(\boldsymbol{\beta}_{0,l}^T\boldsymbol{Z}_j)\boldsymbol{Z}_{\boldsymbol{j}}\}}{E\{\frac{1}{M}\sum_{j=1}^{M} Y_j(t)\exp(\boldsymbol{\beta}_{0,l}^T\boldsymbol{Z}_j)\}},$$

and $\tilde{M}_{ijl}(t;\boldsymbol{\beta}_{0,l},\boldsymbol{\gamma}_0) = \tilde{N}_{ijl}(t;\boldsymbol{\gamma}_0) - \int_0^t Y_{ij}(u)\exp(\boldsymbol{\beta}_{0,l}^T\boldsymbol{Z}_{ij})d\Lambda_{0,l}(u)$, where

$$\Lambda_{0,l}(t) = \int_0^t \frac{E\{\frac{1}{M}\sum_{j=1}^{M} d\tilde{N}_{jl}(u;\boldsymbol{\gamma}_0)\}}{E\{\frac{1}{M}\sum_{j=1}^{M} Y_j(u)\exp(\boldsymbol{\beta}_{0,l}^T\boldsymbol{Z}_j)\}}.$$

The empirical version of $\boldsymbol{\psi}_{ijl}$ is

$$\hat{\boldsymbol{\psi}}_{ijl} = \boldsymbol{H}_{n,l}^{-1}(\hat{\boldsymbol{\beta}}_{n,l};\hat{\boldsymbol{\gamma}}_n)\int_0^{\tau}\{\boldsymbol{Z}_{ij} - \boldsymbol{E}_n(t,\hat{\boldsymbol{\beta}}_{n,l})\}d\hat{M}_{ijl}(t;\hat{\boldsymbol{\beta}}_{n,l},\hat{\boldsymbol{\gamma}}_n),$$

where

$$\boldsymbol{H}_{n,l}(\hat{\boldsymbol{\beta}}_{n,l};\hat{\boldsymbol{\gamma}}_n) = \frac{1}{n}\sum_{i=1}^{n}\frac{1}{M_i}\sum_{j=1}^{M_i}\int_0^{\tau}\boldsymbol{V}_{n,l}(t,\hat{\boldsymbol{\beta}}_{n,l})d\tilde{N}_{ijl}(t;\hat{\boldsymbol{\gamma}}_n),$$

$$\boldsymbol{V}_{n,l}(t,\hat{\boldsymbol{\beta}}_{n,l}) = \frac{\sum_{p=1}^{n}\frac{1}{M_p}\sum_{q=1}^{M_p} Y_{pq}(t)\exp(\hat{\boldsymbol{\beta}}_{n,l}^T\boldsymbol{Z}_{pq})\boldsymbol{Z}_{pq}^{\otimes 2}}{\sum_{p=1}^{n}\frac{1}{M_p}\sum_{q=1}^{M_p} Y_{pq}(t)\exp(\hat{\boldsymbol{\beta}}_{n,l}^T\boldsymbol{Z}_{pq})} - \left\{\frac{\sum_{p=1}^{n}\frac{1}{M_p}\sum_{q=1}^{M_p} Y_{pq}(t)\exp(\hat{\boldsymbol{\beta}}_{n,l}^T\boldsymbol{Z}_{pq})\boldsymbol{Z}_{pq}}{\sum_{p=1}^{n}\frac{1}{M_p}\sum_{q=1}^{M_p} Y_{pq}(t)\exp(\hat{\boldsymbol{\beta}}_{n,l}^T\boldsymbol{Z}_{pq})}\right\}^{\otimes 2},$$

$$\boldsymbol{E}_n(t,\hat{\boldsymbol{\beta}}_{n,l}) = \frac{\sum_{p=1}^{n}\frac{1}{M_p}\sum_{q=1}^{M_p} Y_{pq}(t)\exp(\hat{\boldsymbol{\beta}}_{n,l}^T\boldsymbol{Z}_{pq})\boldsymbol{Z}_{pq}}{\sum_{p=1}^{n}\frac{1}{M_p}\sum_{q=1}^{M_p} Y_{pq}(t)\exp(\hat{\boldsymbol{\beta}}_{n,l}^T\boldsymbol{Z}_{pq})},$$

and

$$\hat{M}_{ijl}(t;\hat{\boldsymbol{\beta}}_{n,l},\hat{\boldsymbol{\gamma}}_n) = \tilde{N}_{ijl}(t;\hat{\boldsymbol{\gamma}}_n) - \int_0^t Y_{ij}(u)\exp(\hat{\boldsymbol{\beta}}_{n,l}^T\boldsymbol{Z}_{ij})d\hat{\Lambda}_{n,l}(u).$$

Also, define the non-random quantity

$$\boldsymbol{R}_l = \boldsymbol{H}_l^{-1}(\boldsymbol{\beta}_{0,l})E\left[\frac{1}{M}\sum_{j=1}^{M}(1-R_j)\int_0^{\tau}\{\boldsymbol{Z}_j - \boldsymbol{E}(t,\boldsymbol{\beta}_{0,l})\}dN_j(t)\dot{\pi}_l(\boldsymbol{W}_j,\boldsymbol{\gamma}_0)^T\right],$$

where $\dot{\pi}_l(\boldsymbol{W}_j,\boldsymbol{\gamma}_0) = \partial\{\pi_l(\boldsymbol{W}_j,\boldsymbol{\gamma})\}(\partial\boldsymbol{\gamma})^{-1}|_{\boldsymbol{\gamma}=\boldsymbol{\gamma}_0}$. The empirical version of $\boldsymbol{R}_l$ is

$$\hat{\boldsymbol{R}}_l = \boldsymbol{H}_{n,l}^{-1}(\hat{\boldsymbol{\beta}}_{n,l};\hat{\boldsymbol{\gamma}}_n)\frac{1}{n}\sum_{i=1}^{n}\left[\frac{1}{M_i}\sum_{j=1}^{M_i}(1-R_{ij})\int_0^{\tau}\{\boldsymbol{Z}_{ij} - \boldsymbol{E}_n(t,\hat{\boldsymbol{\beta}}_{n,l})\}dN_{ij}(t)\dot{\pi}_l(\boldsymbol{W}_{ij},\hat{\boldsymbol{\gamma}}_n)^T\right].$$

Next, for Theorem 2.3, define

$$\phi_{ijl}(t) = \int_0^t \frac{d\tilde{M}_{ijl}(s;\boldsymbol{\beta}_{0,j},\boldsymbol{\gamma}_0)}{E\{\frac{1}{M}\sum_{j=1}^{M} Y_j(s)\exp(\boldsymbol{\beta}_{0,l}^T\boldsymbol{Z}_j)\}} - (\boldsymbol{\psi}_{ijl} + \boldsymbol{R}_l\boldsymbol{\omega}_{ij})^T\int_0^t \boldsymbol{E}(s,\boldsymbol{\beta}_{0,l})d\Lambda_{0,l}(s),$$

and the non-random function

$$\boldsymbol{R}_j^\star(t) = E\left[\frac{1}{M}\sum_{j=1}^{M}(1-R_j)\dot{\pi}_j(\boldsymbol{W}_j,\boldsymbol{\gamma}_0)\int_0^t \frac{dN_j(s)}{E\{\frac{1}{M}\sum_{j=1}^{M}Y_j(s)\exp(\boldsymbol{\beta}_{0,l}^T\boldsymbol{Z}_j)\}}\right]^T.$$

The empirical versions of the latter functions are

$$\hat{\phi}_{ijl}(t) = \int_0^t \frac{d\hat{M}_{ijl}(t;\hat{\boldsymbol{\beta}}_{n,l},\hat{\boldsymbol{\gamma}}_n)}{\frac{1}{n}\sum_{i=1}^{n}\{\frac{1}{M_i}\sum_{j=1}^{M_i}Y_{ij}(s)\exp(\hat{\boldsymbol{\beta}}_{n,l}^T\boldsymbol{Z}_{ij})\}} - (\hat{\boldsymbol{\psi}}_{ijl}+\hat{\boldsymbol{R}}_l\hat{\boldsymbol{\omega}}_{ij})^T\int_0^t \boldsymbol{E}_n(s,\hat{\boldsymbol{\beta}}_{n,l})d\hat{\Lambda}_{n,l}(s),$$

and

$$\hat{\boldsymbol{R}}_l^*(t) = \frac{1}{n}\sum_{i=1}^{n}\left[\frac{1}{M}\sum_{j=1}^{M}(1-R_{ij})\dot{\pi}_j(\boldsymbol{W}_{ij},\hat{\boldsymbol{\gamma}}_n)\int_0^t \frac{dN_{ij}(s)}{\frac{1}{n}\sum_{i=1}^{n}\{\frac{1}{M}\sum_{j=1}^{M}Y_{ij}(s)\exp(\hat{\boldsymbol{\beta}}_{n,l}^T\boldsymbol{Z}_{ij})\}}\right]^T,$$

respectively.

Finally, for Theorem 2.4, define the influence function

$$\begin{aligned}\phi_{ijl}^F(t;\boldsymbol{z}_0) &= \int_0^t \exp\left\{-\sum_{l=1}^{k}\Lambda_{0,l}(s-;\boldsymbol{z}_0)\right\}d\phi_{ijl}^\Lambda(s;\boldsymbol{z}_0)\\ &\quad - \int_0^t\left\{\sum_{l=1}^{k}\phi_{ijl}^\Lambda(s-;\boldsymbol{z}_0)\right\}\exp\left\{-\sum_{l=1}^{k}\Lambda_{0,l}(s-;\boldsymbol{z}_0)\right\}d\Lambda_{0,l}(s;\boldsymbol{z}_0),\end{aligned}$$

where

$$\phi_{ijl}^\Lambda(t;\boldsymbol{z}_0) = \{\boldsymbol{z}_0^T(\boldsymbol{\psi}_{ijl}+\boldsymbol{R}_l\boldsymbol{\omega}_{ij})\Lambda_{0,l}(t)+\phi_{ijl}(t)+\boldsymbol{R}_l^\star(t)\boldsymbol{\omega}_{ij}\}\exp(\boldsymbol{\beta}_{0,l}^T\boldsymbol{z}_0).$$

The empirical version of $\phi_{ijl}^F(t;\boldsymbol{z}_0)$ is

$$\begin{aligned}\hat{\phi}_{ijl}^F(t;\boldsymbol{z}_0) &= \int_0^t \exp\left\{-\sum_{l=1}^{k}\hat{\Lambda}_{n,l}(s-;\boldsymbol{z}_0)\right\}d\hat{\phi}_{ijl}^\Lambda(s;\boldsymbol{z}_0)\\ &\quad - \int_0^t\left\{\sum_{l=1}^{k}\hat{\phi}_{ijl}^\Lambda(s-;\boldsymbol{z}_0)\right\}\exp\left\{-\sum_{l=1}^{k}\hat{\Lambda}_{n,l}(s-;\boldsymbol{z}_0)\right\}d\hat{\Lambda}_{n,l}(s;\boldsymbol{z}_0),\end{aligned}$$

where

$$\hat{\phi}_{ijl}^\Lambda(t;\boldsymbol{z}_0) = \{\boldsymbol{z}_0^T(\hat{\boldsymbol{\psi}}_{ijl}+\hat{\boldsymbol{R}}_l\hat{\boldsymbol{\omega}}_{ij})\hat{\Lambda}_{n,l}(t)+\hat{\phi}_{ijl}(t)+\hat{\boldsymbol{R}}_l^\star(t)\hat{\boldsymbol{\omega}}_{ij}\}\exp(\hat{\boldsymbol{\beta}}_{n,l}^T\boldsymbol{z}_0).$$

2.1 *Proof of Theorem 2.1*

To prove the consistency of $\hat{\boldsymbol{\beta}}_{n,l}$ we use the consistency conditions for general Z-estimators (Kosorok, 2008). In light of condition C5, the population analog of the partial pseudoscore func-

tion is

$$\boldsymbol{G}_l(\boldsymbol{\beta}) = P\left[\frac{1}{M}\sum_{j=1}^{m_0} I(M \geqslant j)\int_0^\tau \{\boldsymbol{Z}_j - \boldsymbol{E}(t,\boldsymbol{\beta})\}d\tilde{N}_{jl}(t;\boldsymbol{\gamma}_0)\right].$$

By conditions C5 and C7 we have

$$\begin{aligned}
\boldsymbol{G}_l(\boldsymbol{\beta}) &= E\left(\frac{1}{M}\sum_{j=1}^{m_0} I(M \geqslant j)E\left[\int_0^\tau \{\boldsymbol{Z}_j - \boldsymbol{E}(t,\boldsymbol{\beta})\}d\tilde{N}_{jl}(t;\boldsymbol{\gamma}_0)\Big| M\right]\right)\\
&= E\left(E\left[\int_0^\tau \{\boldsymbol{Z}_1 - \boldsymbol{E}(t,\boldsymbol{\beta})\}d\tilde{N}_{1l}(t,\boldsymbol{\gamma}_0)\Big| M\right]\frac{1}{M}\sum_{j=1}^{m_0} I(M \geqslant j)\right)\\
&= E\left(E\left[\int_0^\tau \{\boldsymbol{Z}_j - \boldsymbol{E}(t,\boldsymbol{\beta})\}d\tilde{N}_{jl}(t;\boldsymbol{\gamma}_0)\Big| M\right]\right)\\
&= E\left[\int_0^\tau \{\boldsymbol{Z}_j - \boldsymbol{E}(t,\boldsymbol{\beta})\}d\tilde{N}_{jl}(t;\boldsymbol{\gamma}_0)\right], \qquad (2.1)
\end{aligned}$$

for any $j = 1, \ldots, M$. Now, by the definition of $\tilde{N}_{ijl}(t;\boldsymbol{\gamma}_0)$, the assumption of correct specification of the marginal model $\pi_l(\boldsymbol{W}_{ij},\boldsymbol{\gamma}_0)$, and the fact that $\Delta_{ijl}N_{ij}(t) = N_{ijl}(t)$, it follows that

$$\boldsymbol{G}_l(\boldsymbol{\beta}) = E\left[\int_0^\tau \{\boldsymbol{Z}_j - \boldsymbol{E}(t,\boldsymbol{\beta})\}dN_{jl}(t)\right],$$

for any $j = 1, \ldots, M$. Letting $\Lambda_{0,l}(t) = \int_0^t \lambda_{0,l}(s)ds$, $\boldsymbol{G}_l(\boldsymbol{\beta}_{0,l})$ can be expressed as

$$\begin{aligned}
\boldsymbol{G}_l(\boldsymbol{\beta}_{0,l}) &= E\left[\int_0^\tau \{\boldsymbol{Z}_j - \boldsymbol{E}(t,\boldsymbol{\beta}_{0,l})\}d\{N_{jl}(t) - Y_j(t)\exp(\boldsymbol{\beta}_{0,l}^T\boldsymbol{Z}_j)d\Lambda_{0,l}(t)\}\right]\\
&\quad + E\left[\int_0^\tau \{\boldsymbol{Z}_j - \boldsymbol{E}(t,\boldsymbol{\beta}_{0,l})\}Y_j(t)\exp(\boldsymbol{\beta}_{0,l}^T\boldsymbol{Z}_j)d\Lambda_{0,l}(t)\right]. \qquad (2.2)
\end{aligned}$$

Note that, by the independent censoring assumption conditionally on $\boldsymbol{Z}_{ij}$ and under the assumed marginal propotional cause-specific hazards model, we have

$$\begin{aligned}
E\{N_{jl}(t)|\boldsymbol{Z}_j\} &= P(X_j \leqslant t, C_j = l|\boldsymbol{Z}_j)\\
&= P(U_j \geqslant T_j, T_j \leqslant t, C_j = l|\boldsymbol{Z}_j)\\
&= \int_0^t P(U_j \geqslant s|\boldsymbol{Z}_j)dF_l(s;\boldsymbol{Z}_j)\\
&= \int_0^t P(U_j \geqslant s|\boldsymbol{Z}_j)P(T_j > s|\boldsymbol{Z}_j)\frac{dF_l(s;\boldsymbol{Z}_j)}{P(T_j > s|\boldsymbol{Z}_j)}\\
&= \int_0^t P(X_j \geqslant s|\boldsymbol{Z}_j)\exp(\boldsymbol{\beta}_{0,l}^T\boldsymbol{Z}_j)d\Lambda_{0,l}(s)\\
&= E\left\{\int_0^t Y_j(s)\exp(\boldsymbol{\beta}_{0,l}^T\boldsymbol{Z}_j)d\Lambda_{0,l}(s)\Big|\boldsymbol{Z}_j\right\} \qquad (2.3)
\end{aligned}$$

for any $j = 1, \ldots, M$. Also, by conditions C5 and C7 and similar calculations to those in (2.1), we have that

$$\boldsymbol{E}(t, \boldsymbol{\beta}_{0,l}) = \frac{E\left\{Y_j(t)\exp(\boldsymbol{\beta}^T \boldsymbol{Z}_j)\boldsymbol{Z}_j\right\}}{E\left\{Y_j(t)\exp(\boldsymbol{\beta}^T \boldsymbol{Z}_j)\right\}}.$$

This fact, equality (2.3), and some algebra lead to the conclusion that

$$E\left[\int_0^\tau \{\boldsymbol{Z}_j - \boldsymbol{E}(t, \boldsymbol{\beta}_{0,l})\} d\{N_{jl}(t) - Y_j(t)\exp(\boldsymbol{\beta}_{0,l}^T \boldsymbol{Z}_j) d\Lambda_{0,l}(t)\}\right] = \boldsymbol{0}.$$

and

$$E\left[\int_0^\tau \{\boldsymbol{Z}_j - \boldsymbol{E}(t, \boldsymbol{\beta}_{0,l})\} Y_j(t)\exp(\boldsymbol{\beta}_{0,l}^T \boldsymbol{Z}_j) d\Lambda_{0,l}(t)\right] = \boldsymbol{0}.$$

Therefore, by (2.2), it follows that $\boldsymbol{G}_l(\boldsymbol{\beta}_{0,l}) = \boldsymbol{0}$. Condition C6 implies that $\boldsymbol{\beta}_{0,l}$ is the unique root of $\boldsymbol{G}_l(\boldsymbol{\beta}) = \boldsymbol{0}$, $\boldsymbol{\beta} \in \mathcal{B}_l$. To complete the proof of the strong consistency of $\hat{\boldsymbol{\beta}}_{n,l}$, it remains to show that

$$\sup_{\boldsymbol{\beta} \in \mathcal{B}_l} \|\boldsymbol{G}_{n,l}(\boldsymbol{\beta}, \hat{\boldsymbol{\gamma}}_n) - \boldsymbol{G}_l(\boldsymbol{\beta})\| \to_{as*} 0.$$

Using empirical process notation, the empirical version of the pseudoscore function defined in Section 2.2 can be written as

$$\boldsymbol{G}_{n,l}(\boldsymbol{\beta}, \hat{\boldsymbol{\gamma}}_n) = \mathbb{P}_n\left[\frac{1}{M}\sum_{j=1}^{m_0}\int_0^\tau I(M \geqslant j)\{\boldsymbol{Z}_j - \boldsymbol{E}_n(t, \boldsymbol{\beta})\} d\tilde{N}_{jl}(t; \hat{\boldsymbol{\gamma}}_n)\right].$$

The difference between the empirical partial pseudoscore function and expected partial pseudoscore function can be decomposed as

$$\boldsymbol{G}_{n,l}(\boldsymbol{\beta}, \hat{\boldsymbol{\gamma}}_n) - \boldsymbol{G}_l(\boldsymbol{\beta}) = \boldsymbol{A}_{n,l} + \boldsymbol{B}_{n,l} - \boldsymbol{C}_{n,l}(\boldsymbol{\beta}) - \boldsymbol{D}_{n,l}(\boldsymbol{\beta}) - \boldsymbol{E}_{n,l}(\boldsymbol{\beta}),$$

where

$$\boldsymbol{A}_{n,l} = \mathbb{P}_n\left[\frac{1}{M}\sum_{j=1}^{m_0}\int_0^\tau I(M \geqslant j)\boldsymbol{Z}_j\{d\tilde{N}_{jl}(t; \hat{\boldsymbol{\gamma}}_n) - d\tilde{N}_{jl}(t; \boldsymbol{\gamma}_0)\}\right],$$

$$\boldsymbol{B}_{n,l} = (\mathbb{P}_n - P)\left\{\frac{1}{M}\sum_{j=1}^{m_0}\int_0^\tau I(M \geqslant j)\boldsymbol{Z}_j d\tilde{N}_{jl}(t; \boldsymbol{\gamma}_0)\right\},$$

$$\boldsymbol{C}_{n,l}(\boldsymbol{\beta}) = \mathbb{P}_n\left[\frac{1}{M}\sum_{j=1}^{m_0}\int_0^\tau I(M \geqslant j)\boldsymbol{E}_n(t,\boldsymbol{\beta})\{d\tilde{N}_{jl}(t;\hat{\boldsymbol{\gamma}}_n) - d\tilde{N}_{jl}(t;\boldsymbol{\gamma}_0)\}\right],$$

$$\boldsymbol{D}_{n,l}(\boldsymbol{\beta}) = \mathbb{P}_n\left[\frac{1}{M}\sum_{j=1}^{m_0}\int_0^\tau I(M \geqslant j)\{\boldsymbol{E}_n(t,\boldsymbol{\beta}) - \boldsymbol{E}(t,\boldsymbol{\beta})\}d\tilde{N}_{jl}(t;\boldsymbol{\gamma}_0)\right],$$

and

$$\boldsymbol{E}_{n,l}(\boldsymbol{\beta}) = (\mathbb{P}_n - P)\left\{\frac{1}{M}\sum_{j=1}^{m_0}\int_0^\tau I(M \geqslant j)\boldsymbol{E}(t,\boldsymbol{\beta})d\tilde{N}_{jl}(t;\boldsymbol{\gamma}_0)\right\}.$$

By conditions C3-C5,

$$\begin{aligned}
\|\boldsymbol{A}_{n,l}\| &= \left\|\mathbb{P}_n\left[\frac{1}{M}\sum_{j=1}^{m_0}\int_0^\tau I(M \geqslant j)\boldsymbol{Z}_j\{d\tilde{N}_{jl}(t;\hat{\boldsymbol{\gamma}}_n) - d\tilde{N}_{jl}(t;\boldsymbol{\gamma}_0)\}\right]\right\| \\
&= \left\|\mathbb{P}_n\left[\frac{1}{M}\sum_{j=1}^{m_0}\int_0^\tau I(M \geqslant j)\boldsymbol{Z}_j(1-R_j)\{\pi_l(\boldsymbol{W}_j,\hat{\boldsymbol{\gamma}}_n) - \pi_l(\boldsymbol{W}_j,\boldsymbol{\gamma}_0)\}dN_j(t)\right]\right\| \\
&\leqslant K_0\|\hat{\boldsymbol{\gamma}}_n - \boldsymbol{\gamma}_0\|\left\|\mathbb{P}_n\left\{\frac{1}{M}\sum_{j=1}^{m_0}\int_0^\tau I(M \geqslant j)\boldsymbol{Z}_j(1-R_j)dN_j(t)\right\}\right\| \\
&\rightarrow_{as} \boldsymbol{0}.
\end{aligned}$$

By the strong law of large numbers and condition C5,

$$\boldsymbol{B}_{n,l} = (\mathbb{P}_n - P)\left\{\frac{1}{M}\sum_{j=1}^{m_0}\int_0^\tau I(M \geqslant j)\boldsymbol{Z}_j d\tilde{N}_{jl}(t;\boldsymbol{\gamma}_0)\right\} \rightarrow_{as} \boldsymbol{0}.$$

By conditions C2-C5,

$$\begin{aligned}
\sup_{\boldsymbol{\beta}\in\mathcal{B}_l}\|\boldsymbol{C}_{n,l}(\boldsymbol{\beta})\| &= \sup_{\boldsymbol{\beta}\in\mathcal{B}_l}\left\|\mathbb{P}_n\left[\frac{1}{M}\sum_{j=1}^{m_0}\int_0^\tau I(M \geqslant j)\boldsymbol{E}_n(t,\boldsymbol{\beta})\{d\tilde{N}_{jl}(t;\hat{\boldsymbol{\gamma}}_n) - d\tilde{N}_{jl}(t;\boldsymbol{\gamma}_0)\}\right]\right\| \\
&\leqslant \sup_{\boldsymbol{\beta}\in\mathcal{B}_l}\left\|\mathbb{P}_n\left[\frac{1}{M}\sum_{j=1}^{m_0}\int_0^\tau I(M \geqslant j)\boldsymbol{E}_n(t,\boldsymbol{\beta})\{\pi_l(\boldsymbol{W}_j,\hat{\boldsymbol{\gamma}}_n) - \pi_l(\boldsymbol{W}_j,\boldsymbol{\gamma}_0)\}dN_j(t)\right]\right\| \\
&\leqslant K_0\,\|\hat{\boldsymbol{\gamma}}_n - \boldsymbol{\gamma}_0\|\sup_{\boldsymbol{\beta}\in\mathcal{B}_l}\left\|\mathbb{P}_n\left\{\frac{1}{M}\sum_{j=1}^{m_0}\int_0^\tau I(M \geqslant j)\boldsymbol{E}_n(t,\boldsymbol{\beta})dN_j(t)\right\}\right\| \\
&\rightarrow_{as^*} 0.
\end{aligned}$$

For $\boldsymbol{D}_{n,l}(\boldsymbol{\beta})$, the classes of functions $\{M^{-1}\sum_{j=1}^{m_0} I(M \geqslant j)Y_j(t)\exp(\boldsymbol{\beta}^T\boldsymbol{Z}_j), t \in [0,\tau], \boldsymbol{\beta} \in \mathcal{B}_l\}$ and $\{M^{-1}\sum_{j=1}^{m_0} I(M \geqslant j)Y_j(t)\exp(\boldsymbol{\beta}^T\boldsymbol{Z}_j)\boldsymbol{Z}_j, t \in [0,\tau], \boldsymbol{\beta} \in \mathcal{B}_l\}$ are Donsker by condition C5

and, thus, also Glivenko-Cantelli. Therefore, using conditions C2 and C5,

$$
\begin{aligned}
\sup_{\boldsymbol{\beta}\in\mathcal{B}_l} \|\boldsymbol{D}_{n,l}(\boldsymbol{\beta})\| &= \sup_{\boldsymbol{\beta}\in\mathcal{B}_l} \left\| \mathbb{P}_n \left[\frac{1}{M}\sum_{j=1}^{m_0} \int_0^\tau I(M \geqslant j)\{\boldsymbol{E}_n(t,\boldsymbol{\beta}) - \boldsymbol{E}(t,\boldsymbol{\beta})\} d\tilde{N}_{jl}(t;\boldsymbol{\gamma}_0) \right] \right\| \\
&\leqslant \sup_{t\in[0,\tau],\boldsymbol{\beta}\in\mathcal{B}_l} \|\boldsymbol{E}_n(t,\boldsymbol{\beta}) - \boldsymbol{E}(t,\boldsymbol{\beta})\| \sup_{\boldsymbol{\beta}\in\mathcal{B}_l} \left\| \mathbb{P}_n \left\{ \frac{1}{M}\sum_{j=1}^{m_0} \int_0^\tau I(M \geqslant j) d\tilde{N}_{ji}(t;\boldsymbol{\gamma}_0) \right\} \right\| \\
&\leqslant K_0 \sup_{t\in[0,\tau],\boldsymbol{\beta}_l\in\mathcal{B}_l} \|\boldsymbol{E}_n(t,\boldsymbol{\beta}) - \boldsymbol{E}(t,\boldsymbol{\beta})\| \\
&\rightarrow_{as^*} 0.
\end{aligned}
$$

For $\boldsymbol{E}_{n,l}(\boldsymbol{\beta}_l)$, consider the class of functions

$$
\begin{aligned}
\mathcal{L}_l^{(p)} &= \left\{ \frac{1}{M}\sum_{j=1}^{m_0} \int_0^\tau I(M \geqslant j)\boldsymbol{E}^{(p)}(t,\boldsymbol{\beta}) d\tilde{N}_{jl}(t;\boldsymbol{\gamma}_0), \boldsymbol{\beta} \in \mathcal{B}_l \right\} \\
&= \left\{ \frac{1}{M}\sum_{j=1}^{m_0} \{R_j\Delta_{jt} + (1-R_j)\pi_l(\boldsymbol{W}_j,\boldsymbol{\gamma}_0)\} \int_0^\tau I(M \geqslant j) E^{(p)}(t,\boldsymbol{\beta}) dN_j(t), \boldsymbol{\beta} \in \mathcal{B}_l \right\},
\end{aligned}
$$

where

$$
E^{(p)}(t,\boldsymbol{\beta}) = \frac{E\{\frac{1}{M}\sum_{j=1}^{m_0} I(M \geqslant j) Y_j(t) \exp(\boldsymbol{\beta}^T \boldsymbol{Z}_j) Z_j^{(p)}\}}{E\{\frac{1}{M}\sum_{j=1}^{m_0} I(M \geqslant j) Y_j(t) \exp(\boldsymbol{\beta}^T \boldsymbol{Z}_j)\}},
$$

with $Z_j^{(p)}$ being the pth component of $\boldsymbol{Z}_j$. For an arbitrary probability measure Q, define the norm $\|f\|_{Q,2} = (\int f^2 dQ)^{1/2}$. Now, for any finitely discrete probability measure Q and $\forall \boldsymbol{\beta}_1$, $\boldsymbol{\beta}_2 \in \mathcal{B}_l$ and $f_{\boldsymbol{\beta}_1}^{(p)}, f_{\boldsymbol{\beta}_2}^{(p)} \in \mathcal{L}_l^{(p)}$

$$
\begin{aligned}
&\left\| f_{\boldsymbol{\beta}_1}^{(p)} - f_{\boldsymbol{\beta}_2}^{(p)} \right\|_{Q,2} \\
&\leqslant \left\| \frac{1}{M}\sum_{j=1}^{m_0} \{R_j\Delta_{jl} + (1-R_j)\pi_l(\boldsymbol{W}_j,\boldsymbol{\gamma}_0)\} \int_0^\tau I(M \geqslant j) |E^{(p)}(t,\boldsymbol{\beta}_1) - E^{(p)}(t,\boldsymbol{\beta}_2)| dN_j(t) \right\|_{Q,2} \\
&\leqslant \left\| \frac{1}{M}\sum_{j=1}^{m_0} \int_0^\tau |E^{(p)}(t,\boldsymbol{\beta}_1) - E^{(p)}(t,\boldsymbol{\beta}_2)| dN_j(t) \right\|_{Q,2} \\
&\leqslant K_0 \|\boldsymbol{\beta}_1 - \boldsymbol{\beta}_2\|,
\end{aligned}
$$

by the Lipschitz continuity of $E^{(p)}(t,\boldsymbol{\beta})$ and condition C5. Then for $\forall \boldsymbol{\beta} \in \mathcal{B}_l$ there exists a $\boldsymbol{\beta}_i$, $i = 1, \ldots, N(\epsilon, \mathcal{B}_l, \|\cdot\|)$ such that $\|\boldsymbol{\beta}_i - \boldsymbol{\beta}\| < \epsilon$. Therefore, $\forall f_{\boldsymbol{\beta}}^{(p)} \in \mathcal{L}_l^{(p)}$ there exists a $f_{\boldsymbol{\beta}_i}^{(p)}$ such

that

$$\|f^{(p)}_{\boldsymbol{\beta}_i} - f^{(p)}_{\boldsymbol{\beta}}\|_{Q,2} \leqslant K_0\|\boldsymbol{\beta}_i - \boldsymbol{\beta}\| \equiv \epsilon'.$$

Therefore, $\mathcal{L}_l^{(p)}$ can be covered by $N(\epsilon, \mathcal{B}_l, \|\cdot\|)$ $L_2(Q)$ ϵ'-balls centered at $f^{(p)}_{\boldsymbol{\beta}_i}$. Thus, the covering number for $\mathcal{L}_l^{(p)}$ is $N(\epsilon', \mathcal{L}_l^{(p)}, L_2(Q)) \leqslant N(\epsilon, \mathcal{B}_l, \|\cdot\|)$, where $\mathcal{B}_l$ is a Donsker class as a consequence of condition C2. In addition, using similar arguments to those in page 142 in Kosorok (2008), the class $\mathcal{L}_l^{(p)}$ is pointwise measurable. Consequently, $\mathcal{L}_l^{(p)}$ is Donsker and, thus, also Glivenko-Cantelli. Therefore, $\sup_{\boldsymbol{\beta}\in\mathcal{B}_l} \|\boldsymbol{E}_{n,l}(\boldsymbol{\beta})\| \rightarrow_{as^*} 0$ and, thus,

$$\sup_{\boldsymbol{\beta}\in\mathcal{B}_l} \|\boldsymbol{G}_{n,l}(\boldsymbol{\beta}, \hat{\boldsymbol{\gamma}}_n) - \boldsymbol{G}_l(\boldsymbol{\beta})\| \rightarrow_{as^*} 0.$$

This concludes the proof that $\|\hat{\boldsymbol{\beta}}_{n,l} - \boldsymbol{\beta}_{0,l}\| \rightarrow_{as^*} 0$.

Next, we prove the uniform consistency of $\hat{\Lambda}_1(t)$. We define the supremum norm $\|f(t)\|_\infty = \sup_{t\in[0,\tau]} |f(t)|$. Equality (2.3), conditions C5 and C7, and calculations similar to those in (2.1) lead to the conclusion that

$$\Lambda_{0,l}(t) = \int_0^t \frac{P\{\frac{1}{M}\sum_{j=1}^M d\tilde{N}_{jl}(t;\boldsymbol{\gamma}_0)\}}{P\{\frac{1}{M}\sum_{j=1}^M Y_j(t)\exp(\boldsymbol{\beta}_{0,l}^T\boldsymbol{Z}_j)\}}.$$

Now, after some algebra, we have that

$$\hat{\Lambda}_{n,l}(t) - \Lambda_{0,l}(t) = A^*_{n,l}(t) + B^*_{n,l}(t), \tag{2.4}$$

where

$$A^*_{n,l}(t) = \mathbb{P}_n\left[\frac{1}{M}\sum_{j=1}^M (1-R_j)\{\pi_l(\boldsymbol{W}_j, \hat{\boldsymbol{\gamma}}_n) - \pi_l(\boldsymbol{W}_j, \boldsymbol{\gamma}_0)\}\int_0^t \frac{dN_j(t)}{\mathbb{P}_n\{\frac{1}{M}\sum_{j=1}^M Y_j(t)\exp(\hat{\boldsymbol{\beta}}_{n,l}^T\boldsymbol{Z}_j)\}}\right],$$

$$B^*_{n,l}(t) = \left[\int_0^t \frac{\mathbb{P}_n\{\frac{1}{M}\sum_{j=1}^M d\tilde{N}_{jl}(t;\boldsymbol{\gamma}_0)\}}{\mathbb{P}_n\{\frac{1}{M}\sum_{j=1}^M Y_j(t)\exp(\hat{\boldsymbol{\beta}}_{n,l}^T\boldsymbol{Z}_j)\}} - \int_0^t \frac{P\{\frac{1}{M}\sum_{j=1}^M d\tilde{N}_{jl}(t;\boldsymbol{\gamma}_0)\}}{P\{\frac{1}{M}\sum_{j=1}^M Y_j(t)\exp(\boldsymbol{\beta}_{0,l}^T\boldsymbol{Z}_j)\}}\right].$$

By conditions C3, C4 and C5, $\|A^*_{n,l}(\cdot)\|_\infty \rightarrow_{as^*} 0$. By a similar expansion and arguments in Kosorok (2008), $\|B^*_{n,l}(\cdot)\|_\infty \rightarrow_{as^*} 0$. Therefore, $\|\hat{\Lambda}_{n,l} - \Lambda_{0,l}\|_\infty \rightarrow_{as^*} 0$.

2.2 *Proof of Theorem 2.2*

The estimator $\hat{\boldsymbol{\beta}}_{n,l}$ satisfies

$$\begin{aligned}\mathbf{0} &= \sqrt{n}\boldsymbol{G}_{n,l}(\hat{\boldsymbol{\beta}}_{n,l}, \hat{\boldsymbol{\gamma}}_n) \\ &= \sqrt{n}\{\boldsymbol{G}_{n,l}(\hat{\boldsymbol{\beta}}_{n,l}, \hat{\boldsymbol{\gamma}}_n) - \boldsymbol{G}_{n,l}(\hat{\boldsymbol{\beta}}_{n,l}, \boldsymbol{\gamma}_0)\} + \sqrt{n}\boldsymbol{G}_{n,l}(\hat{\boldsymbol{\beta}}_{n,l}, \boldsymbol{\gamma}_0). \qquad (2.5)\end{aligned}$$

The first term in the right side of (2.5) can be expressed as

$$\sqrt{n}\{\boldsymbol{G}_{n,l}(\hat{\boldsymbol{\beta}}_{n,l}, \hat{\boldsymbol{\gamma}}_n) - \boldsymbol{G}_{n,l}(\hat{\boldsymbol{\beta}}_{n,l}, \boldsymbol{\gamma}_0)\} = \boldsymbol{A}'_{n,l} - \boldsymbol{B}'_{n,l} - \boldsymbol{C}'_{n,l} + \boldsymbol{D}'_{n,l},$$

where

$$\boldsymbol{A}'_{n,l} = \sqrt{n}(\mathbb{P}_n - P)\left[\frac{1}{M}\sum_{j=1}^{m_0} I(M \geqslant j)\boldsymbol{Z}_j N_j(\tau)(1 - R_j)\{\pi_l(\boldsymbol{W}_j, \hat{\boldsymbol{\gamma}}_n) - \pi_l(\boldsymbol{W}_j, \boldsymbol{\gamma}_0)\}\right],$$

$$\boldsymbol{B}'_{n,l} = \sqrt{n}(\mathbb{P}_n - P)\left[\frac{1}{M}\sum_{j=1}^{m_0} I(M \geqslant j)(1 - R_j)\{\pi_l(\boldsymbol{W}_j, \hat{\boldsymbol{\gamma}}_n) - \pi_l(\boldsymbol{W}_j, \boldsymbol{\gamma}_0)\}\int_0^\tau \boldsymbol{E}(t, \boldsymbol{\beta}_{0,l})dN_j(t)\right],$$

$$\boldsymbol{C}'_{n,l} = \sqrt{n}(\mathbb{P}_n - P)\left[\frac{1}{M}\sum_{j=1}^{m_0}\int_0^\tau I(M \geqslant j)\{\boldsymbol{E}_n(t, \boldsymbol{\beta}_l) - \boldsymbol{E}(t, \boldsymbol{\beta}_{0,l})\}d\{\tilde{N}_{jl}(t; \hat{\boldsymbol{\gamma}}_n) - \tilde{N}_{jl}(t; \boldsymbol{\gamma}_0)\}\right],$$

and

$$\boldsymbol{D}'_{n,l} = P\left[\frac{1}{M}\sum_{j=1}^{m_0} I(M \geqslant j)(1 - R_j)\int_0^\tau \{\boldsymbol{Z}_j - \boldsymbol{E}_n(t, \boldsymbol{\beta}_{0,l})\}dN_j(t)\dot{\pi}_l(\boldsymbol{W}_j, \boldsymbol{\gamma}_0)^T\right] \times \sqrt{n}(\hat{\boldsymbol{\gamma}}_n - \boldsymbol{\gamma}_0).$$

By conditions C3, C5 and the continuous mapping theorem it follows that $\boldsymbol{A}'_{n,l} \to_p \mathbf{0}$ and $\boldsymbol{B}'_{n,l} \to_p \mathbf{0}$. Using Lemma 4.2 of Kosorok (2008), it follows that $\boldsymbol{C}'_{n,l} \to_p \mathbf{0}$. Therefore, the first term in the right side of (2.5) is $\sqrt{n}\{\boldsymbol{G}_{n,l}(\hat{\boldsymbol{\beta}}_{n,l}, \hat{\boldsymbol{\gamma}}_n) - \boldsymbol{G}_{n,l}(\hat{\boldsymbol{\beta}}_{n,l}, \boldsymbol{\gamma}_0)\} = \boldsymbol{A}'_{n,l} - \boldsymbol{B}'_{n,l} - \boldsymbol{C}'_{n,l} + \boldsymbol{D}'_{n,l} = \boldsymbol{D}'_{n,l} + o_p(1)$. The second term in the right side of (2.5) can be expressed as

$$\sqrt{n}\boldsymbol{G}_{n,l}(\hat{\boldsymbol{\beta}}_{n,l}, \boldsymbol{\gamma}_0) = \sqrt{n}\boldsymbol{G}_{n,l}(\boldsymbol{\beta}_{0,l}, \boldsymbol{\gamma}_0) - \boldsymbol{H}_l(\boldsymbol{\beta}_{0,l})\sqrt{n}(\hat{\boldsymbol{\beta}}_{n,l} - \boldsymbol{\beta}_{0,l}) + o_p(1 + \sqrt{n}\|\hat{\boldsymbol{\beta}}_{n,l} - \boldsymbol{\beta}_{0,l}\|).$$

By condition C6, $\boldsymbol{H}_l(\boldsymbol{\beta}_{0,l})$ is invertible and, thus, there exists a constant $K_0 > 0$ such that for any $\boldsymbol{\beta}_l \in \mathcal{B}_l$, $\|\boldsymbol{H}_l(\boldsymbol{\beta}_{0,l})(\boldsymbol{\beta}_l - \boldsymbol{\beta}_{0,l})\| \geqslant K_0\|\boldsymbol{\beta}_l - \boldsymbol{\beta}_{0,l}\|$. Therefore, by Taylor expansion around $\boldsymbol{\beta}_{0,l}$,

$\|\boldsymbol{G}_{n,l}(\boldsymbol{\beta}_l, \boldsymbol{\gamma}_0) - \boldsymbol{G}_l(\boldsymbol{\beta}_{0,l}, \boldsymbol{\gamma}_0)\| \geqslant K_0\|\boldsymbol{\beta}_l - \boldsymbol{\beta}_{0,l}\| + o_p(\|\boldsymbol{\beta}_l - \boldsymbol{\beta}_{0,l}\|)$. Now,

$$\begin{aligned}
&\sqrt{n}\}\boldsymbol{G}_{n,l}(\hat{\boldsymbol{\beta}}_{n,l}, \boldsymbol{\gamma}_0) - \boldsymbol{G}_l(\boldsymbol{\beta}_{0,l}, \boldsymbol{\gamma}_0)\} \\
&= \sqrt{n}(\mathbb{P}_n - P)\left[\frac{1}{M}\sum_{j=1}^{m_0}\int_0^\tau I(M \geqslant j)\{\boldsymbol{Z}_j - E(t, \boldsymbol{\beta}_{0,l})\}d\tilde{N}_{jl}(t; \boldsymbol{\gamma}_0)\right] \\
&\quad + o_p(1 + \sqrt{n}\|\boldsymbol{\beta}_{n,l} - \boldsymbol{\beta}_{0,l}\|) + o_p(1) \\
&= O_p(1) + o_p(1 + \sqrt{n}\|\hat{\boldsymbol{\beta}}_{n,l} - \boldsymbol{\beta}_{0,l}\|) + o_p(1).
\end{aligned}$$

Consequently, $\{K_0 + o_p(1)\}\sqrt{n}\|\hat{\boldsymbol{\beta}}_{n,l} - \boldsymbol{\beta}_{0,l}\| \leqslant O_p(1) + o_p(1 + \sqrt{n}\|\hat{\boldsymbol{\beta}}_{n,l} - \boldsymbol{\beta}_{0,l}\|) + o_p(1)$, and thus $\sqrt{n}\|\hat{\boldsymbol{\beta}}_{n,l} - \boldsymbol{\beta}_{0,l}\| = O_p(1)$. This leads to the conclusion that $\sqrt{n}\boldsymbol{G}_{n,l}(\hat{\boldsymbol{\beta}}_{n,l}, \boldsymbol{\gamma}_0) = \sqrt{n}\boldsymbol{G}_{n,l}(\boldsymbol{\beta}_{0,l}, \boldsymbol{\gamma}_0) - \boldsymbol{H}_l(\boldsymbol{\beta}_{0,l})\sqrt{n}(\hat{\boldsymbol{\beta}}_{n,l} - \boldsymbol{\beta}_{0,l}) + o_p(1)$. Recalling that $\tilde{M}_{jl}(t; \boldsymbol{\beta}_{0,l}, \boldsymbol{\gamma}_0) = \tilde{N}_{jl}(t; \boldsymbol{\gamma}_0) - \int_0^t I(X_j \geqslant u)\exp(\boldsymbol{\beta}_{0,l}^T\boldsymbol{Z}_j)d\Lambda_{0,l}(u)$ we have

$$\sqrt{n}\boldsymbol{G}_{n,l}(\boldsymbol{\beta}_{0,l}, \boldsymbol{\gamma}_0) = \sqrt{n}\mathbb{P}_n\left[\frac{1}{M}\sum_{j=1}^{m_0}\int_0^\tau I(M \geqslant j)\left\{\boldsymbol{Z}_j - \boldsymbol{E}(t, \boldsymbol{\beta}_{0,l})\right\}d\tilde{M}_{jl}(t; \boldsymbol{\beta}_{0t}, \boldsymbol{\gamma}_0)\right] + o_p(1).$$

Taking all the pieces together we have that

$$\begin{aligned}
\mathbf{0} &= \sqrt{n}\boldsymbol{G}_{n,l}(\hat{\boldsymbol{\beta}}_{n,l}, \hat{\boldsymbol{\gamma}}_n) \\
&= \sqrt{n}\{\boldsymbol{G}_{n,l}(\hat{\boldsymbol{\beta}}_{n,l}, \hat{\boldsymbol{\gamma}}_n) - \boldsymbol{G}_{n,l}(\hat{\boldsymbol{\beta}}_{n,l}, \boldsymbol{\gamma}_0)\} + \sqrt{n}\boldsymbol{G}_{n,l}(\hat{\boldsymbol{\beta}}_{n,l}, \boldsymbol{\gamma}_0) \\
&= P\left\{\frac{1}{M}\sum_{j=1}^{m_0} I(M \geqslant j)(1 - R_j)\int_0^\tau [\boldsymbol{Z}_j - \boldsymbol{E}_n(t, \boldsymbol{\beta}_{0,l})]dN_j(t)\dot{\pi}_l(\boldsymbol{W}_j, \boldsymbol{\gamma}_0)^T\right\} \times \sqrt{n}(\hat{\boldsymbol{\gamma}}_n - \boldsymbol{\gamma}_0) \\
&\quad + \sqrt{n}\mathbb{P}_n\left\{\frac{1}{M}\sum_{j=1}^{m_0}\int_0^\tau I(M \geqslant j)\left\{\boldsymbol{Z}_j - \boldsymbol{E}(t, \boldsymbol{\beta}_{0,l})\right\}d\tilde{M}_{jl}(t; \boldsymbol{\beta}_{0t}, \boldsymbol{\gamma}_0)\right\} \\
&\quad - \boldsymbol{H}_l(\boldsymbol{\beta}_{0,l})\sqrt{n}(\hat{\boldsymbol{\beta}}_{n,l} - \boldsymbol{\beta}_{0,l}) + o_p(1).
\end{aligned}$$

Rearranging the terms and according to conditions C4 and C6 leads to

$$\sqrt{n}(\hat{\boldsymbol{\beta}}_{n,l} - \boldsymbol{\beta}_{0,l}) = \frac{1}{\sqrt{n}}\sum_{i=1}^n\left\{\frac{1}{M_i}\sum_{j=1}^{M_i}(\boldsymbol{\psi}_{ijl} + \boldsymbol{R}_l\boldsymbol{\omega}_{ij})\right\} + o_p(1),$$

where

$$\boldsymbol{\psi}_{ijl} = \boldsymbol{H}_l^{-1}(\boldsymbol{\beta}_{0,l})\int_0^\tau\{\boldsymbol{Z}_{ij} - \boldsymbol{E}(t, \boldsymbol{\beta}_{0,l})\}d\tilde{M}_{ijl}(t; \boldsymbol{\beta}_{0,l}, \boldsymbol{\gamma}_0),$$

and

$$R_l = \boldsymbol{H}_l^{-1}(\boldsymbol{\beta}_{0,l})E\left[\frac{1}{M}\sum_{j=1}^{M}(1-R_j)\int_0^{\tau}\{\boldsymbol{Z}_j - \boldsymbol{E}(t,\boldsymbol{\beta}_{0,l})\}dN_j(t)\dot{\pi}_l(\boldsymbol{W}_j,\boldsymbol{\gamma}_0)^T\right].$$

2.3 *Proof of Theorem 2.3*

By Taylor expansion and the consistency of $\hat{\boldsymbol{\beta}}_{n,l}$ and $\hat{\boldsymbol{\gamma}}_n$, the first term in the right side of expansion (2.4) can be written as

$$\begin{aligned}A^*_{n,l}(t) &= \mathbb{P}_n\left[\frac{1}{M}\sum_{j=1}^{M}(1-R_j)\{\pi_l(\boldsymbol{W}_j,\hat{\boldsymbol{\gamma}}_n) - \pi_l(\boldsymbol{W}_j,\boldsymbol{\gamma}_0)\}\int_0^t \frac{dN_j(t)}{\mathbb{P}_n\{\frac{1}{M}\sum_{j=1}^{M}Y_j(t)\exp(\hat{\boldsymbol{\beta}}_{n,l}^T\boldsymbol{Z}_j)\}}\right]\\ &= \frac{1}{\sqrt{n}}\sum_{i=1}^{n}\left\{\frac{1}{M_i}\sum_{j=1}^{M_i}\boldsymbol{R}_l^*(t)\boldsymbol{\omega}_{ij}\right\} + o_p(n^{-1/2}),\end{aligned}$$

where

$$\boldsymbol{R}_j^{\star}(t) = E\left[\frac{1}{M}\sum_{j=1}^{M}(1-R_j)\dot{\pi}_j(\boldsymbol{W}_j,\boldsymbol{\gamma}_0)\int_0^t\frac{dN_j(s)}{E\{\frac{1}{M}\sum_{j=1}^{M}Y_j(s)\exp(\boldsymbol{\beta}_{0,l}^T\boldsymbol{Z}_j)\}}\right]^T.$$

By similar analysis to that provided in Page 57 of Kosorok (2008), the second term in (2.4) can be written as

$$\begin{aligned}B^*_{n,l}(t) &= \left[\int_0^t\frac{\mathbb{P}_n\{\frac{1}{M}\sum_{j=1}^{M}d\tilde{N}_{jl}(t;\boldsymbol{\gamma}_0)\}}{\mathbb{P}_n\{\frac{1}{M}\sum_{j=1}^{M}Y_j(t)\exp(\hat{\boldsymbol{\beta}}_{n,l}^T\boldsymbol{Z}_j)\}} - \int_0^t\frac{P\{\frac{1}{M}\sum_{j=1}^{M}d\tilde{N}_{jl}(t;\boldsymbol{\gamma}_0)\}}{P\{\frac{1}{M}\sum_{j=1}^{M}Y_j(t)\exp(\boldsymbol{\beta}_{0,l}^T\boldsymbol{Z}_j)\}}\right]\\ &= \frac{1}{\sqrt{n}}\sum_{i=1}^{n}\left\{\frac{1}{M_i}\sum_{j=1}^{M_i}\phi_{ijl}(t)\right\} + o_p(n^{-1/2}),\end{aligned}$$

where

$$\phi_{ijl}(t) = \int_0^t\frac{d\tilde{M}_{ijl}(s;\boldsymbol{\beta}_{0,j},\boldsymbol{\gamma}_0)}{E\{\frac{1}{M}\sum_{j=1}^{M}Y_j(s)\exp(\boldsymbol{\beta}_{0,l}^T\boldsymbol{Z}_j)\}} - (\boldsymbol{\psi}_{ijl} + \boldsymbol{R}_l\boldsymbol{\omega}_{ij})^T\int_0^t E(s,\boldsymbol{\beta}_{0,l})d\Lambda_{0,l}(s).$$

Therefore,

$$\sqrt{n}\left\{\hat{\Lambda}_{n,l}(t) - \Lambda_{0,l}(t)\right\} = \frac{1}{\sqrt{n}}\sum_{i=1}^{n}\left[\frac{1}{M_i}\sum_{j=1}^{M_i}\{\phi_{ijl}(t) + \boldsymbol{R}_l^*(t)\boldsymbol{\omega}_{ij}\}\right] + o_p(1).$$

By conditions C1, C4, and C5, and lemmas 1 and 2 in the supporting information of Bakoyannis (2021), the class of functions

$$\left[\frac{1}{M}\sum_{j=1}^{M}\{\phi_{jl}(t)+\boldsymbol{R}_l^*(t)\boldsymbol{\omega}_j\} : t\in[0,\tau]\right]$$

is Donsker. We now show that conditional on the data, the estimated multiplier process

$$\hat{W}_{n,l}(\cdot)=\frac{1}{\sqrt{n}}\sum_{i=1}^{n}\left[\frac{1}{M_i}\sum_{j=1}^{M_i}\{\hat{\phi}_{ijl}(\cdot)+\hat{\boldsymbol{R}}_l^*(\cdot)\hat{\boldsymbol{\omega}}_{ij}\}\right]\xi_i$$

converges weakly to the same limiting process as $W_{n,l}(\cdot)=\sqrt{n}\{\hat{\Lambda}_{n,l}(\cdot)-\Lambda_{0,l}(\cdot)\}$. Define

$$\tilde{W}_{n,l}(\cdot)=\frac{1}{\sqrt{n}}\sum_{i=1}^{n}\left[\frac{1}{M_i}\sum_{j=1}^{M_i}\{\phi_{ijl}(\cdot)+\boldsymbol{R}_l^*(\cdot)\boldsymbol{\omega}_{ij}\}\right]\xi_i.$$

By the Donsker property of the class of influence functions and the conditional multiplier central limit theorem (van der Vaart and Wellner, 1996), $\tilde{W}_{n,l}(\cdot)$ converges weakly, conditionally on the data, to the same limiting process as $W_{n,l}(\cdot)$. To complete the proof, we need to show

$$\|\hat{W}_{n,l}(\cdot)-\tilde{W}_{n,l}(\cdot)\|_\infty=o_p(1),$$

unconditionally. After some algebra

$$\|\hat{W}_{n,l}(\cdot)-\tilde{W}_{n,l}(\cdot)\|_\infty\leqslant A''_{n,l}+B''_{n,l}+C''_{n,l},$$

where

$$A''_{n,l}=\left\|\frac{1}{\sqrt{n}}\sum_{i=1}^{n}\left[\frac{1}{M_i}\sum_{j=1}^{M_i}\left\{\hat{\phi}_{ijl}(\cdot)-\phi_{ijl}(\cdot)\right\}\right]\xi_i\right\|_\infty,$$

$$B''_{n,l}=\sup_{t\in[0,\tau]}\left\|\hat{\boldsymbol{R}}_l^*(t)-\boldsymbol{R}_l^*(t)\right\|\times\left(\left\|\frac{1}{\sqrt{n}}\sum_{i=1}^{n}\left\{\frac{1}{M_i}\sum_{j=1}^{M_i}(\hat{\boldsymbol{\omega}}_{ij}-\boldsymbol{\omega}_{ij})\right\}\xi_i\right\|+\left\|\frac{1}{\sqrt{n}}\sum_{i=1}^{n}\left(\frac{1}{M_i}\sum_{j=1}^{M_i}\boldsymbol{\omega}_{ij}\right)\xi_i\right\|\right),$$

and

$$C''_{n,l}=\sup_{t\in[0,\tau]}\|\boldsymbol{R}_l^*(t)\|\times\left\|\frac{1}{\sqrt{n}}\sum_{i=1}^{n}\left\{\frac{1}{M_i}\sum_{j=1}^{M_i}(\hat{\boldsymbol{\omega}}_{ij}-\boldsymbol{\omega}_{ij})\right\}\xi_i\right\|.$$

Using the same arguments to those used in the proof of Theorem 4 in Spiekerman and Lin (1998) and regularity conditions C3 and C4, $A''_{n,l}=o_p(1)$.

For $B''_{n,l}$, using the same arguments to those used in the proof of Lemma A.3 in Spiekerman and Lin (1998) leads to the conclusion that

$$\left\| \frac{1}{\sqrt{n}} \sum_{i=1}^{n} \left\{ \frac{1}{M_i} \sum_{j=1}^{M_i} (\hat{\boldsymbol{\omega}}_{ij} - \boldsymbol{\omega}_{ij}) \right\} \xi_i \right\| = o_p(1).$$

Next, by condition C4 and the central limit theorem

$$\left\| \frac{1}{\sqrt{n}} \sum_{i=1}^{n} \left(\frac{1}{M_i} \sum_{j=1}^{M_i} \boldsymbol{\omega}_{ij} \right) \xi_i \right\| = O_p(1).$$

Also, after some algebra, we have that

$$\sup_{t\in[0,\tau]} \left\| \hat{\boldsymbol{R}}_l^*(t) - \boldsymbol{R}_l^*(t) \right\| \leqslant A'''_{n,l} + B'''_{n,l} + C'''_{n,l},$$

where

$$A'''_{n,l} = \sup_{t\in[0,\tau]} \left\| \mathbb{P}_n \left[\frac{1}{M} \sum_{j=1}^{M} \{ \dot{\pi}_l(\boldsymbol{W}_j, \hat{\gamma}_n) - \dot{\pi}_l(\boldsymbol{W}_j, \boldsymbol{\gamma}_0) \} \int_0^t \frac{dN_j(s)}{\mathbb{P}_n \left\{ \frac{1}{M} \sum_{j=1}^{M} Y_j(s) \exp\left(\hat{\boldsymbol{\beta}}_{n,l}^T \boldsymbol{Z}_j \right) \right\}} \right] \right\|,$$

$$B'''_{n,l} = \sup_{t\in[0,\tau]} \left\| \mathbb{P}_n \frac{1}{M} \sum_{j=1}^{M} \dot{\pi}_l(\boldsymbol{W}_j, \boldsymbol{\gamma}_0) \int_0^t \left[\frac{1}{\mathbb{P}_n \{ \frac{1}{M} \sum_{j=1}^{M} Y_j(s) e^{\hat{\boldsymbol{\beta}}_{n,l}^T \boldsymbol{Z}_j} \}} - \frac{1}{P \{ \frac{1}{M} \sum_{j=1}^{M} Y_j(s) e^{\boldsymbol{\beta}_{0,l}^T \boldsymbol{Z}_j} \}} \right] dN_j(s) \right\|,$$

and

$$C'''_{n,l} = \sup_{t\in[0,\tau]} \left\| (\mathbb{P}_n - P) \left[\frac{1}{M} \sum_{j=1}^{M} \dot{\pi}_l(\boldsymbol{W}_j, \boldsymbol{\gamma}_0) \int_0^t \frac{1}{P \left\{ \frac{1}{M} \sum_{j=1}^{M} Y_j(s) \exp\left(\boldsymbol{\beta}_{0,l}^T \boldsymbol{Z}_j \right) \right\}} dN_j(s) \right] \right\|.$$

By conditions C3, C4, C5, and the continuous mapping theorem,

$$\max_{ij} \| \dot{\pi}_l(\boldsymbol{W}_{ij}, \hat{\gamma}_n) - \dot{\pi}_l(\boldsymbol{W}_{ij}, \boldsymbol{\gamma}_0) \| = o_{as}(1).$$

By Theorem 2.2 and conditions C1, C2, and C5,

$$\left\| \frac{1}{\mathbb{P}_n \left\{ \frac{1}{M} \sum_{j=1}^{M} Y_j(\cdot) \exp(\hat{\boldsymbol{\beta}}_{n,l}^T \boldsymbol{Z}_j) \right\}} \right\|_\infty = \left\| \frac{1}{P \left\{ \frac{1}{M} \sum_{j=1}^{M} Y_j(\cdot) \exp(\boldsymbol{\beta}_{0,l}^T \boldsymbol{Z}_j) \right\} + o_{as^*}(1)} \right\|_\infty = O_{as^*}(1).$$

Therefore, $A'''_{n,l} = o_{as^*}(1)$. Next, by conditions C3 and C5, $\max_{ij} \| \dot{\pi}_l(\boldsymbol{W}_{ij}, \boldsymbol{\gamma}_0) \| = O_{as}(1)$. Also, by conditions C2, C5, and the Donsker property of $\{Y_j(t) : t \in [0,\tau]\}$,

$$\left\| \frac{1}{\mathbb{P}_n \left\{ \frac{1}{M} \sum_{j=1}^{M} Y_j(\cdot) \exp\left(\hat{\boldsymbol{\beta}}_{n,l}^T \boldsymbol{Z}_j \right) \right\}} - \frac{1}{P \left\{ \frac{1}{M} \sum_{j=1}^{M} Y_j(\cdot) \exp\left(\boldsymbol{\beta}_{0,l}^T \boldsymbol{Z}_j \right) \right\}} \right\|_\infty = o_{as^*}(1),$$

and, thus, $B'''_{n,l} = o_{as^*}(1)$. For $C'''_{n,l}$, consider the classes of functions $\mathcal{F} = \{N_j(s) : t \in [0,\tau]\}$ and

$$\mathcal{L}_{l,1} = \left\{ f_{t,l} = \frac{1}{M}\sum_{j=1}^{m_0} I(M \geqslant j)\dot{\pi}_l(\boldsymbol{W}_j, \boldsymbol{\gamma}_0)\int_0^t \frac{1}{P\{\frac{1}{M}\sum_{j=1}^M Y_j(s)e^{\boldsymbol{\beta}_{0,l}^T \boldsymbol{Z}_j}\}} dN_j(s), t \in [0,\tau] \right\}.$$

For any finitely discrete probability measure Q and any t_1, $t_2 \in [0,\tau]$ we have that

$$\begin{aligned}\|f_{t_1,l} - f_{t_2,l}\|_{Q,2} &\leqslant \left\| \frac{1}{M}\sum_{j=1}^{m_0} I(M \geqslant j)\dot{\pi}_l(\boldsymbol{W}_j, \boldsymbol{\gamma}_0)\int_{t_1}^{t_2} \frac{dN_j(s)}{P\{\frac{1}{M}\sum_{j=1}^M Y_j(s)\exp(\boldsymbol{\beta}_{0,l}^T \boldsymbol{Z}_j)\}} \right\|_{Q,2} \\ &\leqslant K_0 \|N_j(t_2) - N_j(t_1)\|_{Q,2}.\end{aligned}$$

Therefore, $\mathcal{L}_{l,1}$ can be covered by $N(\epsilon, \mathcal{F}, L_2(Q))$ $L_2(Q)$ ϵ' -balls centered at $f_{t_i,l}$, where $\mathcal{F}$ is a Donsker class by lemma 4.1 in Kosorok (2008). In addition, using similar arguments to those in page 142 in Kosorok (2008), the class $\mathcal{L}_{l,1}$ is pointwise measurable. Consequently, the class $\mathcal{L}_{l,1}$ is Donsker and, thus, also Glivenko-Cantelli, which leads to the conclusion that $C'''_{n,l} = o_{as^*}(1)$. Therefore, $\sup_{t\in[0,\tau]} \|\hat{\boldsymbol{R}}_l^*(t) - \boldsymbol{R}_l^*(t)\| = o_p(1)$ and, thus. $B''_{n,l} = o_p(1)$. Similar arguments lead to the conclusion that $C''_{n,l} = o_p(1)$. Thus,

$$\left\|\hat{W}_{n,l}(\cdot) - \tilde{W}_{n,l}(\cdot)\right\|_\infty = o_p(1),$$

which completes the proof of the last statement in Theorem 2.3.

2.4 *Proof of Theorem 2.4*

It is easy to show that

$$\begin{aligned}\tilde{W}_{n,l}(t;\boldsymbol{z}_0) &= \sqrt{n}\{\hat{\Lambda}_{n,l}(t;\boldsymbol{z}_0) - \Lambda_{0,l}(t;\boldsymbol{z}_0)\} \\ &= \sqrt{n}\{\hat{\Lambda}_{n,l}(t)\exp(\hat{\boldsymbol{\beta}}_{n,l}^T\boldsymbol{z}_0) - \Lambda_{0,l}(t)\exp(\boldsymbol{\beta}_{0,l}^T\boldsymbol{z}_0)\} \\ &= \sqrt{n}\{\hat{\Lambda}_{n,l}(t) - \Lambda_{0,l}(t) + \boldsymbol{z}_0^T(\hat{\boldsymbol{\beta}}_{n,l} - \boldsymbol{\beta}_{0,l})\Lambda_{0,l}(t)\}\exp(\boldsymbol{\beta}_{0,l}^T\boldsymbol{z}_0) + o_p(1) \\ &= \frac{1}{\sqrt{n}}\sum_{i=1}^n \frac{1}{M_i}\sum_{j=1}^{M_i}\{\boldsymbol{z}_0^T(\boldsymbol{\psi}_{ijl} + \boldsymbol{R}_l\boldsymbol{\omega}_{ij})\Lambda_{0,l}(t) + \phi_{ijl}(t) + \boldsymbol{R}_l^*(t)\boldsymbol{\omega}_{ij}\}\exp(\boldsymbol{\beta}_{0,l}^T\boldsymbol{z}_0) + o_p(1) \\ &= \frac{1}{\sqrt{n}}\sum_{i=1}^n \frac{1}{M_i}\sum_{j=1}^{M_i}\phi_{ijl}^{\Lambda}(t;\boldsymbol{z}_0) + o_p(1).\end{aligned}$$

Similarly to the decomposition in Cheng *and others* (1998),

$$\sqrt{n}\left\{\hat{F}_{n,l}(t;\boldsymbol{z}_0)-F_{0,l}(t;\boldsymbol{z}_0)\right\}=\frac{1}{\sqrt{n}}\sum_{i=1}^{n}\left\{\frac{1}{M_i}\sum_{j=1}^{M_i}\phi_{ijl}^{F}(t;\boldsymbol{z}_0)\right\}+o_p(1),$$

where

$$\phi_{ijl}^{F}(t;\boldsymbol{z}_0)=\int_0^t \exp\left\{-\sum_{l=1}^{k}\Lambda_{0,l}(s-;\boldsymbol{z}_0)\right\}d\phi_{ijl}^{\Lambda}(s;\boldsymbol{z}_0)$$
$$-\int_0^t\left\{\sum_{l=1}^{k}\phi_{ijl}^{\Lambda}(s-;\boldsymbol{z}_0)\right\}\exp\left\{-\sum_{l=1}^{k}\Lambda_{0,l}(s-;\boldsymbol{z}_0)\right\}d\Lambda_{0,l}(s;\boldsymbol{z}_0),$$

and $\phi_{ijl}^{\Lambda}(t;\boldsymbol{z}_0)=\{\boldsymbol{z}_0^T(\boldsymbol{\psi}_{ijl}+\boldsymbol{R}_l\boldsymbol{\omega}_{ij})\Lambda_{0,l}(t)+\phi_{ijl}(t)+\boldsymbol{R}_l^{\star}(t)\boldsymbol{\omega}_{ij}\}\exp(\boldsymbol{\beta}_{0,l}^T\boldsymbol{z}_0)$. The class of functions $\{\phi_{jl}^{F}(t;\boldsymbol{z}_0:t\in[0,\tau]\}$ is Donsker by conditions C1, C4, and C5, lemmas 1 and 2 in the supporting information of Bakoyannis (2021), and corollary 9.32 in Kosorok (2008).

To conclude the proof of Theorem 2.4, we show that, conditionally on the data,

$$\hat{W}_{n,l}^{F}(\cdot;\boldsymbol{z}_0)=\frac{1}{\sqrt{n}}\sum_{i=1}^{n}\frac{1}{M_i}\sum_{j=1}^{M_i}\hat{\phi}_{ijl}^{F}(\cdot;\boldsymbol{z}_0)\xi_i$$

converges weakly to the same limiting process as $W_{n,l}^{F}(\cdot;\boldsymbol{z}_0)=\sqrt{n}\{\hat{F}_{n,l}(\cdot;\boldsymbol{z}_0)-F_{0,l}(\cdot;\boldsymbol{z}_0)\}$. Now, define

$$\tilde{W}_{n,l}^{F}(\cdot;\boldsymbol{z}_0)=\frac{1}{\sqrt{n}}\sum_{i=1}^{n}\frac{1}{M_i}\sum_{j=1}^{M_i}\phi_{ijl}^{F}(\cdot;\boldsymbol{z}_0)\xi_i.$$

By the Donsker property of the class of influence functions and the conditional multiplier central limit theorem (van der Vaart and Wellner, 1996), $\tilde{W}_{n,l}^{F}(\cdot;\boldsymbol{z}_0)$ converges weakly, conditionally on the data, to the same limiting process as $W_{n,l}^{F}(\cdot;\boldsymbol{z}_0)$. To complete the proof, we need to show that

$$\|\hat{W}_{n,l}^{F}(\cdot;\boldsymbol{z}_0)-\tilde{W}_{n,l}^{F}(\cdot;\boldsymbol{z}_0)\|_\infty=o_p(1),$$

unconditionally. Some algebra leads to the following bound

$$\|\hat{W}_{n,l}^{F}(\cdot;\boldsymbol{z}_0)-\tilde{W}_{n,l}^{F}(\cdot;\boldsymbol{z}_0)\|_\infty\leqslant A_{n,l}''''+B_{n,l}''''+C_{n,l}''''+D_{n,l}''''+E_{n,l}''''+F_{n,l}'''',$$

where

$$A_{n,l}''''=\left\|\int_0^t\exp\left\{-\sum_{l=1}^{k}\hat{\Lambda}_{n,l}\left(\cdot-;\boldsymbol{z}_0\right)\right\}d\left[\sqrt{n}\mathbb{P}_n\frac{1}{M}\sum_{j=1}^{M}\left\{\hat{\phi}_{jl}^{A}\left(\cdot;\boldsymbol{z}_0\right)-\phi_{jl}^{A}\left(\cdot;\boldsymbol{z}_0\right)\right\}\xi\right]\right\|_\infty,$$

$$B''''_{n,l} = \left\| \int_0^t \exp\left\{-\sum_{l=1}^k \hat{\Lambda}_{n,l}(\cdot-;\boldsymbol{z}_0)\right\} - \exp\left\{-\sum_{l=1}^k \Lambda_{0,l}(\cdot-;\boldsymbol{z}_0)\right\} d\left\{\sqrt{n}\mathbb{P}_n \frac{1}{M}\sum_{j=1}^M \phi_{jl}^{\Lambda}(\cdot;\boldsymbol{z}_0)\,\xi\right\}\right\|_\infty,$$

$$C''''_{n,j} = \left\| \sum_{l=1}^k \int_0^t \left[\mathbb{P}_n \tfrac{1}{M}\sum_{j=1}^M \left\{\hat{\phi}_{jl}^A(\cdot-;\boldsymbol{z}_0) - \phi_{jl}^A(\cdot-;\boldsymbol{z}_0)\right\}\xi\right] \exp\left\{-\sum_{l=1}^k \hat{\Lambda}_{n,l}(\cdot-;\boldsymbol{z}_0)\right\} \times d\left[\sqrt{n}\left\{\hat{\Lambda}_{n,l}(\cdot;\boldsymbol{z}_0) - A_{0,l}(\cdot;\boldsymbol{z}_0)\right\}\right]\right\|_\infty,$$

$$D''''_{n,j} = \left\| \sum_{l=1}^k \int_0^t \left\{\mathbb{P}_n \tfrac{1}{M}\sum_{j=1}^M \phi_{jl}^A(\cdot-;\boldsymbol{z}_0)\,\xi\right\}\left[\exp\{-\sum_{l=1}^k \hat{\Lambda}_{n,l}(\cdot-;\boldsymbol{z}_0)\} - \exp\{-\sum_{l=1}^k \Lambda_{n,l}(\cdot-;\boldsymbol{z}_0)\}\right] \times d\left[\sqrt{n}\{\hat{\Lambda}_{n,l}(\cdot;\boldsymbol{z}_0) - \Lambda_{0,l}(\cdot;\boldsymbol{z}_0)\}\right]\right\|_\infty,$$

$$E''''_{n,l} = \left\| \sum_{l=1}^k \int_0^t \left[\sqrt{n}\mathbb{P}_n \frac{1}{M}\sum_{j=1}^M \left\{\hat{\phi}_{jl}^A(\cdot-;\boldsymbol{z}_0) - \phi_{jl}^A(\cdot-;\boldsymbol{z}_0)\right\}\xi\right] d\Lambda_{0,l}(\cdot;\boldsymbol{z}_0)\right\|_\infty,$$

$$F''''_{n,l} = O_p(1)\left|\sqrt{n}\mathbb{P}_n\xi\right| \left\| \int_0^t [\exp\{-\sum_{l=1}^k \hat{\Lambda}_{n,l}(\cdot-;\boldsymbol{z}_0)\} - \exp\{-\sum_{l=1}^k \Lambda_{0,l}(\cdot-;\boldsymbol{z}_0)\}] d\Lambda_{0,l}(\cdot;\boldsymbol{z}_0)\right\|_\infty.$$

By integration by parts, Theorem 2.3, Lemma A.3 in Spiekerman and Lin (1998) and the boundedness conditions, $A''''_{n,l} = o_p(1)$. By Theorem 2.1, the Donsker property of the class $\{\phi_{jl}^{\Lambda}(t;\boldsymbol{z}_0) : t \in [0,\tau]\}$, and arguments similar to those used in the proof of proposition 7.27 in Kosorok (2008), $B''''_{n,l} = o_p(1)$. For $C''''_{n,l}$, the integrand converges uniformly to 0 in probability and, thus, by Theorem 2.3 and arguments similar to those used in the proof of proposition 7.27 in Kosorok (2008), it follows that $C''''_{n,l} = o_p(1)$. Using similar arguments, it can be shown that $D''''_{n,l} = o_p(1)$. For $E''''_{n,l}$, the integrand converges uniformly to 0 in probability and, thus, by condition C1 it follows that $E''''_{n,l} = o_p(1)$. Finally, by Theorem 2.1, condition C1, and the central limit theorem, it follows that $F''''_{n,l} = o_p(1)$. Therefore,

$$\left\|\hat{W}_{n,l}^F(\cdot;\boldsymbol{z}_0) - \tilde{W}_{n,l}^F(\cdot;\boldsymbol{z}_0)\right\|_\infty = o_p(1),$$

which completes the proof of the last statement in Theorem 2.4.

3. Additional Simulation Results

The simulation results for the pointwise estimates of the infinite-dimensional parameters $\Lambda_{0,1}(t)$ and $F_{0,1}(t)$ under scenario 1 are provided in Table 1. Simulation results under scenario 2 are

presented in Table 2, 3, and 4.

4. Additional HIV Data Analysis Results

Descriptive characteristics of EA-IeDEA study sample are presented in Table 5. The estimated residual process for the parametric model for the probability of death among those who were lost to clinic

$$\text{logit}\{\pi_1(\boldsymbol{W}, \boldsymbol{\gamma}_0)\} = \gamma_0 + \gamma_1 T + \gamma_2 I(Sex = \text{“}male\text{”}) + \gamma_3 Age + \gamma_4 CD4 + \gamma_5 I(HIV status = \text{“}yes\text{”}),$$

along with the corresponding 95% goodness-of-fit band, are depicted in Figure 1.

References

Bakoyannis, G. (2021). Nonparametric analysis of nonhomogeneous multistate processes with clustered observations. *Biometrics* **77**, 533–546.

Bakoyannis, G., Zhang, Y. and Yiannoutsos, C. T. (2019). Nonparametric inference for Markov processes with missing absorbing state. *Statistica Sinica* **29**, 2083–2104.

Bakoyannis, G., Zhang, Y. and Yiannoutsos, C. T. (2020). Semiparametric regression and risk prediction with competing risks data under missing cause of failure. *Lifetime Data Analysis* **26**, 659–684.

Cheng, S. C., Fine, J. P. and Wei, L. J. (1998). Prediction of cumulative incidence function under the proportional hazards model. *Biometrics* **54**, 219–228.

Kosorok, M. R. (2008). *Introduction to Empirical Processes and Semiparametric Inference.* New York: Springer.

Pan, Z. and Lin, D. Y. (2005). Goodness-of-fit methods for generalized linear mixed models. *Biometrics* **61**, 1000–1009.

Table 1. Simulation results for the infinite-dimensional parameters $\Lambda_{0,1}(t)$ and $F_{0,1}(t)$ at selected time points under Scenario 1. Results from the proposed approach and the approach by Bakoyannis *and others* (2020) (BZY20) which ignores the within-cluster dependence.

			Proposed				BZY20			
n	$p_m(\%)$	t	Bias	MCSD	ASE	CP	Bias	MCSD	ASE	CP
						$\Lambda_{0,1}(t)$				
50	25	0.1	0.001	0.084	0.080	0.915	0.090	0.104	0.025	0.252
		0.2	0.000	0.105	0.101	0.917	0.120	0.127	0.034	0.259
		0.4	0.004	0.135	0.130	0.934	0.160	0.156	0.049	0.284
		0.8	0.011	0.185	0.176	0.947	0.203	0.201	0.081	0.385
	43	0.1	0.000	0.086	0.081	0.915	0.091	0.106	0.031	0.306
		0.2	-0.001	0.108	0.104	0.926	0.121	0.130	0.042	0.318
		0.4	0.003	0.139	0.134	0.935	0.160	0.160	0.058	0.355
		0.8	0.011	0.188	0.181	0.942	0.203	0.203	0.093	0.441
200	25	0.1	0.001	0.040	0.040	0.954	0.090	0.049	0.013	0.074
		0.2	0.001	0.051	0.051	0.951	0.122	0.062	0.017	0.055
		0.4	0.003	0.066	0.066	0.955	0.159	0.077	0.024	0.055
		0.8	0.006	0.093	0.088	0.950	0.200	0.101	0.040	0.096
	43	0.1	0.001	0.040	0.041	0.952	0.091	0.051	0.015	0.096
		0.2	0.002	0.053	0.052	0.952	0.123	0.064	0.021	0.081
		0.4	0.003	0.069	0.068	0.952	0.161	0.079	0.029	0.077
		0.8	0.007	0.096	0.091	0.947	0.199	0.103	0.046	0.138
						$F_{0,1}(t)$				
50	25	0.1	-0.002	0.049	0.047	0.924	0.036	0.055	0.014	0.300
		0.2	-0.003	0.051	0.049	0.926	0.033	0.057	0.015	0.343
		0.4	-0.002	0.053	0.051	0.933	0.025	0.058	0.017	0.388
		0.8	-0.002	0.053	0.051	0.938	0.015	0.058	0.018	0.429
	43	0.1	-0.002	0.049	0.047	0.924	0.036	0.056	0.018	0.381
		0.2	-0.003	0.053	0.051	0.924	0.033	0.059	0.020	0.427
		0.4	-0.002	0.055	0.053	0.934	0.026	0.060	0.023	0.493
		0.8	-0.002	0.056	0.054	0.936	0.015	0.060	0.024	0.555
200	25	0.1	0.000	0.023	0.024	0.954	0.038	0.027	0.007	0.177
		0.2	0.000	0.025	0.025	0.950	0.035	0.028	0.008	0.198
		0.4	0.000	0.026	0.026	0.952	0.028	0.029	0.008	0.287
		0.8	0.000	0.027	0.026	0.945	0.016	0.029	0.009	0.402
	43	0.1	0.000	0.024	0.024	0.952	0.038	0.028	0.009	0.204
		0.2	0.000	0.026	0.026	0.953	0.036	0.029	0.010	0.276
		0.4	0.000	0.028	0.027	0.949	0.028	0.030	0.011	0.368
		0.8	0.000	0.028	0.027	0.945	0.017	0.030	0.012	0.503

Note: n: number of clusters with cluster size $M \in [30, 60]$; p_m: percentage of missingness; t: selected time points ; MCSD: Monte Carlo standard deviation; ASE: average estimated standard error; CP: coverage probability of 95% pointwise confidence interval

Table 2. Simulation results for the regression coefficient β_1 under Scenario 2 for the proposed approach and the approach by Bakoyannis *and others* (2020) (BZY20) which ignores the within-cluster dependence.

		Proposed				BZY20			
n	p_m(%)	Bias	MCSD	ASE	CP	Bias	MCSD	ASE	CP
50	26	-0.002	0.035	0.033	0.938	0.008	0.035	0.023	0.781
	36	0.000	0.036	0.035	0.936	0.011	0.036	0.025	0.803
	44	0.002	0.037	0.036	0.936	0.013	0.037	0.027	0.832
100	26	0.002	0.024	0.024	0.950	0.012	0.023	0.016	0.740
	36	0.004	0.025	0.025	0.937	0.014	0.024	0.018	0.771
	44	0.005	0.026	0.026	0.937	0.016	0.025	0.019	0.782
200	26	0.003	0.017	0.017	0.945	0.013	0.016	0.011	0.681
	36	0.005	0.017	0.018	0.943	0.016	0.017	0.012	0.672
	44	0.007	0.018	0.018	0.940	0.018	0.018	0.014	0.663

Note: n: number of clusters with cluster size $M \in [30, 60]$; p_m: percentage of missingness; MCSD: Monte Carlo standard deviation; ASE: average estimated standard error; CP: coverage probability of 95% confidence interval

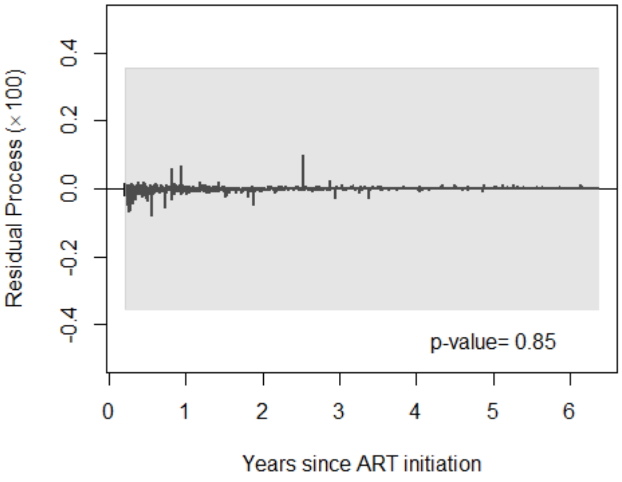

Fig. 1. Plot for the cumulative residual process of the parametric model $\pi_1(\boldsymbol{W}, \boldsymbol{\gamma}_0)$ for death while in care among those lost to clinic based on the HIV data along with the 95% goodness-of-fit band (grey area) and the corresponding p-value.

Table 3. Simulation results for the infinite-dimensional parameters $\Lambda_{0,1}(t)$ and $F_{0,1}(t)$ at selected time points under Scenario 2. Results from the proposed approach and the approach by Bakoyannis *and others* (2020) (BZY20) which ignores the within-cluster dependence.

			Proposed				BZY20			
n	$p_m(\%)$	t	Bias	MCSD	ASE	CP	Bias	MCSD	ASE	CP
						$\Lambda_{0,1}(t)$				
50	26	0.1	-0.004	0.088	0.083	0.911	0.084	0.106	0.028	0.308
		0.2	-0.013	0.109	0.104	0.907	0.104	0.129	0.037	0.317
		0.4	-0.014	0.141	0.134	0.922	0.139	0.161	0.052	0.339
		0.8	0.004	0.193	0.182	0.939	0.200	0.210	0.083	0.405
	44	0.1	-0.008	0.088	0.083	0.907	0.078	0.106	0.034	0.376
		0.2	-0.024	0.110	0.105	0.897	0.090	0.130	0.044	0.403
		0.4	-0.028	0.142	0.136	0.911	0.121	0.162	0.061	0.422
		0.8	-0.001	0.196	0.186	0.945	0.194	0.214	0.095	0.461
200	26	0.1	-0.004	0.041	0.042	0.955	0.085	0.050	0.014	0.101
		0.2	-0.012	0.053	0.052	0.931	0.106	0.062	0.018	0.109
		0.4	-0.015	0.068	0.067	0.934	0.139	0.078	0.026	0.111
		0.8	-0.001	0.096	0.091	0.948	0.196	0.104	0.041	0.111
	44	0.1	-0.007	0.041	0.042	0.942	0.079	0.050	0.017	0.163
		0.2	-0.021	0.053	0.053	0.916	0.092	0.063	0.022	0.201
		0.4	-0.029	0.069	0.068	0.916	0.121	0.079	0.030	0.205
		0.8	-0.006	0.098	0.093	0.940	0.188	0.106	0.047	0.168
						$F_{0,1}(t)$				
50	26	0.1	0.001	0.045	0.043	0.928	0.024	0.051	0.013	0.368
		0.2	-0.002	0.048	0.046	0.925	0.017	0.054	0.015	0.409
		0.4	-0.003	0.051	0.049	0.931	0.008	0.056	0.016	0.425
		0.8	-0.001	0.052	0.050	0.932	-0.002	0.057	0.017	0.448
	44	0.1	0.004	0.046	0.044	0.929	0.026	0.052	0.018	0.452
		0.2	-0.002	0.050	0.048	0.922	0.017	0.055	0.020	0.483
		0.4	-0.004	0.053	0.051	0.927	0.008	0.058	0.022	0.533
		0.8	-0.001	0.054	0.052	0.933	-0.001	0.058	0.023	0.567
200	26	0.1	0.003	0.022	0.022	0.957	0.025	0.025	0.007	0.270
		0.2	0.000	0.024	0.024	0.947	0.019	0.027	0.007	0.347
		0.4	-0.002	0.025	0.025	0.943	0.009	0.028	0.008	0.420
		0.8	-0.001	0.026	0.025	0.944	-0.001	0.029	0.009	0.442
	44	0.1	0.006	0.022	0.023	0.952	0.029	0.026	0.009	0.306
		0.2	0.000	0.025	0.024	0.942	0.019	0.028	0.010	0.434
		0.4	-0.002	0.026	0.026	0.945	0.009	0.029	0.011	0.517
		0.8	0.000	0.027	0.027	0.939	0.000	0.030	0.012	0.554

Note: n: number of clusters with cluster size $M \in [30, 60]$; p_m: percentage of missingness; t: selected time points ;

MCSD: Monte Carlo standard deviation; ASE: average estimated standard error; CP: coverage probability of 95%

pointwise confidence interval

Table 4. Simulation results for the coverage probabilities of 95% simultaneous confidence bands for the infinite-dimensional parameters $\Lambda_{0,1}(t)$ and $F_{0,1}(t)$ under Scenario 2. Results from the proposed approach and the approach by Bakoyannis *and others* (2020) (BZY20) which ignores the within-cluster dependence.

		$\Lambda_{0,1}(t)$				$F_{0,1}(t)$			
n	$p_m(\%)$	Proposed		BZY20		Proposed		BZY20	
		EP	HW	EP	HW	EP	HW	EP	HW
50	26	0.768	0.914	0.078	0.152	0.803	0.924	0.087	0.171
	36	0.684	0.908	0.073	0.166	0.731	0.923	0.081	0.169
	44	0.598	0.915	0.078	0.163	0.661	0.927	0.078	0.172
100	26	0.755	0.942	0.038	0.098	0.796	0.935	0.035	0.113
	36	0.568	0.938	0.026	0.089	0.609	0.931	0.024	0.089
	44	0.421	0.941	0.013	0.069	0.452	0.927	0.010	0.060
200	26	0.684	0.946	0.009	0.045	0.717	0.940	0.013	0.065
	36	0.348	0.942	0.002	0.035	0.358	0.929	0.001	0.034
	44	0.169	0.922	0.000	0.012	0.179	0.914	0.000	0.013

Note: n: number of clusters with cluster size $M \in [30, 60]$; p_m: percentage of missingness; EP: equal precision bands; HW: Hall-Wellner-type bands

Table 5. Descriptive characteristics for the EA-IeDEA study sample

Variable	Right censoring In care (N=8082)	Disengagement (N=3240)	Cause of failure Death (N=1951[1])	Missing (N=11099)
	n (%)	n (%)	n (%)	n (%)
Gender				
Female	5334 (66.0)	2002 (61.8)	974 (49.9)	7362 (66.3)
Male	2748 (34.0)	1238 (38.2)	977 (50.1)	3737 (33.7)
HIV status disclosed				
Yes	5116 (63.3)	2022 (62.4)	1417 (72.6)	6916 (62.3)
No	2966 (36.7)	1218 (37.6)	534 (27.4)	4183 (37.7)
	Median (IQR)	Median (IQR)	Median (IQR)	Median (IQR)
Age[2]	38.0 (31.5, 46.1)	36.1 (29.4, 43.3)	39.1 (32.3, 48.2)	36.0 (29.3, 43.9)
CD4[3]	196 (95, 297)	173 (72, 281)	66 (22, 168)	183 (82, 291)

Note: [1]: Included 84 reported deaths and 1867 unreported deaths confirmed through outreach; [2]: Age at ART initiation in years; [3]: CD4 count at ART initiation in cells/μl

Spiekerman, C. F. and Lin, D. Y. (1998). Marginal regression models for multivariate failure time data. *Journal of the American Statistical Association* **93**, 1164–1175.

van der Vaart, A. W. and Wellner, J. A. (1996). *Weak Convergence and Empirical Processes with Applications to Statistics*. New York: Springer-Verlag.

[*Received August 1, 2010; revised October 1, 2010; accepted for publication November 1, 2010*]
